# Managing cryptic biodiversity: Fine‐scale intralacustrine speciation along a benthic gradient in Alpine whitefish (*Coregonus* spp.)

**DOI:** 10.1111/eva.12446

**Published:** 2016-12-20

**Authors:** Alan G. Hudson, Baenz Lundsgaard‐Hansen, Kay Lucek, Pascal Vonlanthen, Ole Seehausen

**Affiliations:** ^1^ Division of Aquatic Ecology & EvolutionInstitute of Ecology and EvolutionUniversity of BernBernSwitzerland; ^2^ Department of Fish Ecology & EvolutionCentre of Ecology, Evolution and BiogeochemistryEawag Swiss Federal Institute of Aquatic Science and TechnologyKastanienbaumSwitzerland; ^3^ School of Biological SciencesUniversity of BristolBristolUK; ^4^ Department of Animal and Plant SciencesUniversity of SheffieldSheffieldUK; ^5^ Department of Environmental SciencesUniversity of BaselBaselSwitzerland; ^6^ Aquabios GmbH, Les FermesCordastSwitzerland

**Keywords:** adaptive radiation, biodiversity assessment, clinal speciation, *Coregonus*, environmental gradients, fisheries management, speciation‐with‐gene‐flow, stocking

## Abstract

Whitefish (*Coregonus* spp.) are an important catch for many freshwater fisheries, particularly in Switzerland. In support of this, supplemental stocking of whitefish species is carried out, despite lacking complete knowledge of the extent, distribution and origin of whitefish diversity in these lakes, potentially threatening local endemics via artificial gene flow. Here, we investigate phenotypic and genetic differentiation among coexisting whitefish species spawning along a depth gradient in a subalpine Swiss lake to better delineate intralacustrine whitefish biodiversity. We find depth‐related clines in adaptive morphology and in neutral genetic markers. This individual variation is structured in three distinct clusters with spatial overlap. Individual genetic distances correlate strongly with differences in growth rate and gill‐raker number, consistent with predictions of isolation‐by‐adaptation and ecological speciation. Genetic differentiation between species suggests reproductive isolation, despite demographic admixture on spawning grounds. Our results are consistent with clinal speciation resulting in three species coexisting in close ecological parapatry, one (*C*. sp. “benthic intermediate”) being previously unknown. A second unknown species spawning in close proximity was found to be of potential allochthonous origin. This study highlights the importance of taxonomically unbiased sampling strategies to both understand evolutionary mechanisms structuring biodiversity and to better inform conservation and fisheries management.

## 1 Introduction

Theoretical work suggests that speciation is more likely to occur when populations are spatially structured along environmental gradients rather than in complete geographic overlap, as divergent selection regimes encountered at different locations along the gradient act on both phenotypic traits related to ecology and those related to reproductive isolation to reduce homogenizing gene flow (Doebeli & Dieckmann, 2003; Endler, 1977; Gavrilets, 2004; Kawata, Shoji, Kawamura, & Seehausen, 2007; Lande, 1982; Payne, Mazzucco, & Dieckmann, 2011). Given the ubiquity of environmental gradients in nature, clinal speciation along such gradients might be an important generator of new species (Doebeli & Dieckmann, 2003; Gavrilets, 2014). Critical parameters affecting the likelihood of speciation in many models of clinal speciation are the steepness of the environmental gradient relative to dispersal distance, levels of philopatry in mating and the likelihood of behavioural reproductive isolation to evolve (Doebeli & Dieckmann, 2003; Kawata et al., 2007; Lande, 1982; Payne et al., 2011). If assortative mating evolves, clinal speciation can occur under a relatively wide range of model parameters (Doebeli & Dieckmann, 2003; Endler, 1977; Kawata et al., 2007; Lande, 1982). While theoretical support for the importance of environmental gradients in driving speciation is strong, empirical evidence still lags behind (Smith, Wayne, Girman, & Bruford, 1997; Grahame, Wilding, & Butlin, 2006; Seehausen et al., 2008; Magalhaes, Lundsgaard‐Hansen, Mwaiko, & Seehausen, 2012; reviewed by Gavrilets, 2014).

Clinal speciation may be especially important in aquatic environments. In lakes, abiotic factors such as light intensity/composition, oxygen concentration and temperature alongside dependent biotic factors such as the abundance and type of trophic resources, parasites and predators commonly change in a predictable manner: (i) along a depth axis from shallow to deep and (ii) along a benthic–limnetic axis from the sediment surface to open water areas (Seehausen & Wagner, 2014). The higher density of water relative to air also means that individuals in relatively close proximity may experience highly divergent selection regimes, potentially allowing speciation at a finely structured spatial scale, despite high rates of gene flow (Seehausen & Wagner, 2014; Seehausen et al., 2008).

European whitefish (*Coregonus lavaretus* species complex) radiations are excellent systems to empirically investigate mechanisms of speciation along environmental gradients. Numerous lakes across the Palearctic temperate zone harbour multiple coexisting and closely related *Coregonus* species (Hudson, Vonlanthen, Müller, & Seehausen, 2007; Steinmann, 1950; Svärdson, 1979). Many of these radiations comprise evolutionarily young taxa (likely <15,000 years) where reproductive isolation is incomplete and evidence of the mechanisms driving divergence and the key traits involved have not been obscured by postspeciational evolution (Bhat et al., 2014; Coyne & Orr, 2004; Woods, Müller, & Seehausen, 2009). Coexisting whitefish species differ most strongly in phenotypic traits related to foraging and habitat utilization in lacustrine environments such as gill‐raker counts, adult body size and hence growth rate, as well as aspects of body/head shape (Bernatchez, 2004; Harrod, Mallela, & Kahilainen, 2010; Vonlanthen et al., 2009). Also phenotypically similar ecomorphs have arisen independently within many different *Coregonus* radiations, strongly implicating the action of divergent natural selection in the origin of these distinct taxa (Hudson, Vonlanthen, Bezault, & Seehausen, 2013; Hudson, Vonlanthen, & Seehausen, 2011; Østbye, Bernatchez, Næsje, Himberg, & Hindar, 2005; Præbel et al., 2013). Alongside differentiation in habitat use and concomitant trophic ecology, whitefish radiations exhibit parallel patterns of divergence in reproductive ecology. Larger, sparsely gill‐rakered, whitefish tend to spawn at shallower depths than coexisting species of small, densely rakered, zooplanktivorous ecomorphs (Steinmann, 1950; Vonlanthen et al., 2012). Different ecomorphs within lakes also often show temporal differences in the onset and duration of spawning (Steinmann, 1950; Svärdson, 1979). While factors such as lake area, oxygenated depth range, thermal regime and the presence of habitat‐specific predators have been shown to influence both the likelihood of multiple coexisting *Coregonus* species being present, and also the overall whitefish phenotypic and genetic diversity found within a lake (Ingram, Hudson, Vonlanthen, & Seehausen, 2012; Kahilainen, Malinen, & Lehtonen, 2009; Kahilainen, Patterson, Sonninen, Harrod, & Kiljunen, 2014; Landry, Vincent, & Bernatchez, 2007; Siwertsson et al., 2010; Vonlanthen et al., 2012), little research has yet been undertaken to understand how clines in trophic and reproductive ecology interact in the origin of whitefish species and their ongoing coexistence (but see Vonlanthen et al., 2009; Hirsch, Eklov, & Svanbäck, 2013).

**Figure 1:**
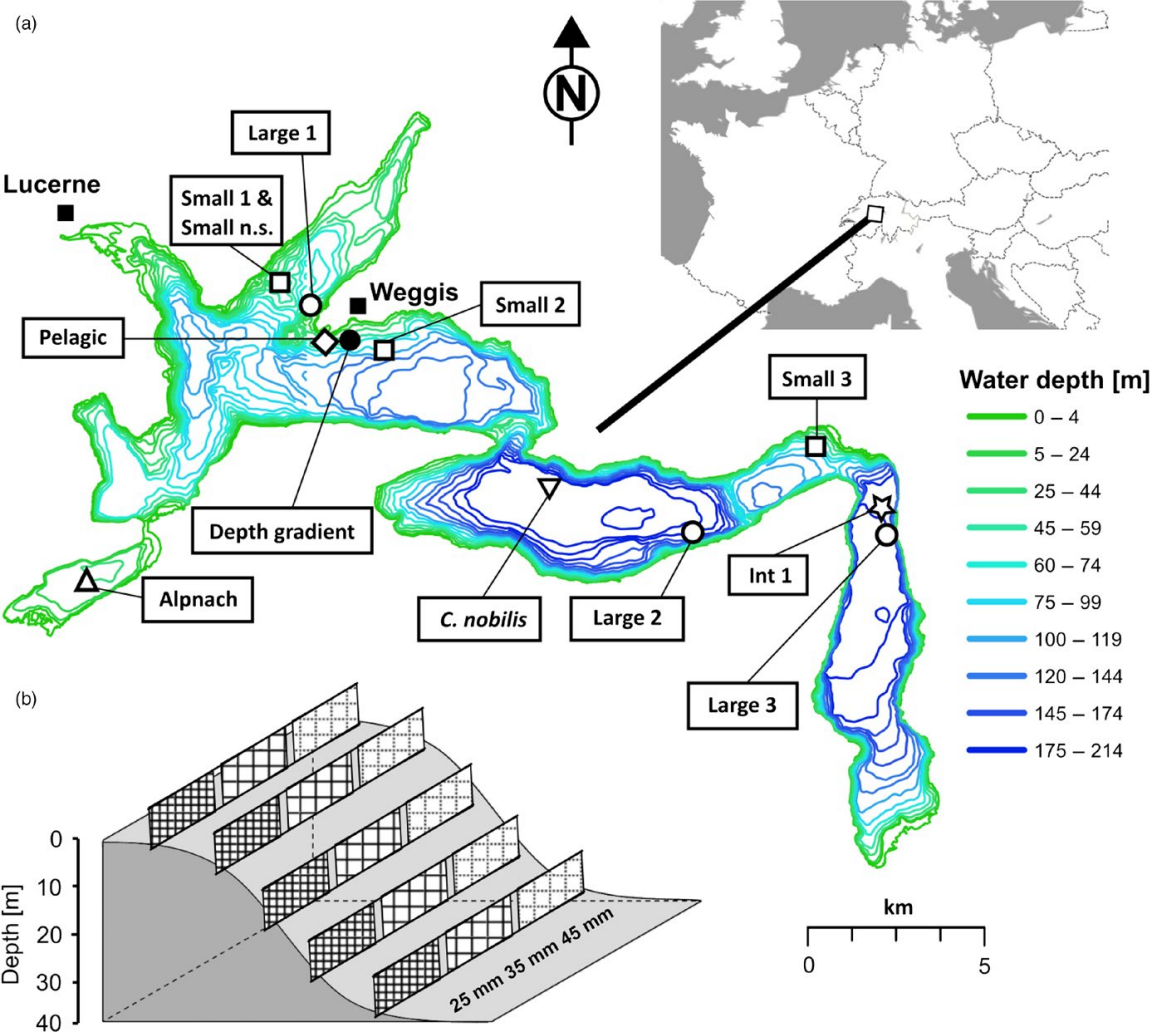
Map of Lake Lucerne. (a) The open circles show the reference sampling sites of the large standard length (SL) class of whitefish, and open squares, the reference sampling locations of the small SL class. The triangle corresponds to whitefish sampled in Lake Alpnach, the reversed triangle to the sampling location of *Coregonus nobilis*, the diamond to whitefish caught in the pelagic and the star to the additional sample of intermediate SL class whitefish. (b) Sampling design for the depth gradient sampling (black circle). Three mesh sizes (25, 35 and 45 mm) were set in five different depths (2, 10, 20, 30 and 40 m) at five different dates covering the spawning time range of all winter‐spawning whitefish species

Around 30 species of whitefish in the larger subalpine lakes of Switzerland have arisen following the glacial retreat ~15,000 years BP (Hudson et al., 2011; Steinmann, 1950; Vonlanthen et al., 2012). These lakes are characterized by high levels of endemicity in their whitefish fauna. Whitefish stocks form an important commercial and cultural resource with supportive breeding carried out during the spawning seasons of the target species. While this supplemental stocking may have helped maintain some native whitefish species experiencing anthropogenic eutrophication in the mid‐to‐late 20th century, overall stocking has negatively impacted endemic diversity. This negative impact has occurred both through the translocation of non‐native whitefish species among lakes and through increased interspecific gene flow via uncontrolled crossing when carrying out supportive breeding (Eckmann, 2012; Hirsch et al., 2013; Hudson et al., 2011). Many of the Alpine lakes harbouring whitefish radiations have recovered from eutrophication (Vonlanthen et al., 2012); however, uncontrolled supportive breeding is still routinely carried out. Given the fragility of evolutionarily young adaptive radiations (Seehausen, 2006), there exists strong potential for conflict between maintaining biodiversity and current fisheries management practice. This potential conflict is exacerbated by the fact that thorough, taxonomically unbiased sampling has not been carried out in many lakes, so the true extent of intralacustrine biodiversity may be underestimated. Currently in Lake Lucerne (Figure 1a), a radiation of three endemic whitefish species is known: the large sparsely rakered winter‐spawning *C*. sp. “Bodenbalchen,” the small and densely rakered summer‐to‐winter‐spawning *Coregonus zugensis*, and the summer‐spawning *Coregonus nobilis* of intermediate size with a rather high number of gill‐rakers (Kottelat & Freyhof, 2007). A fourth winter‐spawning species of intermediate size and gill‐raker number, *C*. sp. “Alpnacherfelchen,” has also been posited, seemingly confined to Lake Alpnach, a large embayment separated by a 300‐m‐long channel from Lake Lucerne (Hudson et al., 2011; Svarvar & Müller, 1982). Individuals not corresponding to the phenotype of any known Lake Lucerne whitefish species have been occasionally reported, suggesting the potential for additional unknown species to reside in Lake Lucerne (Douglas & Brunner, 2002; Steinmann, 1950).

Here, we investigate the distribution and structure of adaptive phenotypic and neutral genetic variation among winter‐spawning whitefish in Lake Lucerne. We sampled individuals continuously throughout the spawning season along a depth gradient from shallow littoral to deep benthic. Variation in functional morphology and genotypic data from 10 microsatellite loci was quantified and used to compare fish caught on the habitat gradient with whitefish of known species assignation, from distinct spawning aggregations across the lake. This allowed the effective delineation of *Coregonus* species diversity with fine‐scale temporal and spatial resolution and to identify key anthropogenic impacts on whitefish diversity within Lake Lucerne. Given the known presence of *C*. sp. “Bodenbalchen” and *C. zugensis* at the extremes of a winter‐spawning depth gradient, we test whether intermediate or indeterminate individuals of whitefish, as reported by fishermen, exist on this gradient and whether these individuals are actually representative of distinct phenotypic and genetic clusters. We subsequently test whether such clusters reflect the known whitefish biodiversity of Lake Lucerne or whether they may constitute additional, previously unknown “cryptic” species. Given the occurrence of anthropogenic stocking among and within Swiss lakes, we then aim to assess the putative origins of the identified clusters, that is whether they represent introduced species or whether they evolved within the native adaptive radiation of Lake Lucerne whitefish.

## 2 Materials and Methods

### 2.1 Sampling

Lake Lucerne (surface area: 114 km^2^) is a deep (maximum depth: 214 m; average depth: 104 m) subalpine lake in central Switzerland within the Reuss river system (Figure 1a). Our sampling design comprised single‐time‐point surveys of whitefish spawning aggregations at different geographic locations throughout the lake, combined with focused fishing of a depth gradient at multiple dates spanning the spawning time/depth range of the two winter‐spawning species that were known from the main lake: *C. zugensis* and *C*. sp. “Bodenbalchen” (Table 1, Figure 1a). Depth gradient fishing was performed using benthic gill nets, each with a surface area of 250 m^2^. To cover the known body size range of the spawning fish, each net was comprised of three panels of separate mesh sizes set in series: 25, 35 and 45 mm. For each of the five sampling date iterations (19 November 2007, 26 November 2007, 05 December 2007, 11 December 2007 and 18 December 2007), nets were set at five different water depths (2, 10, 20, 30 and 40 m) covering the known spawning depths of *C. zugensis* and *C*. sp. “Bodenbalchen,” as well as intermediate depths (Figure 1b, these samples are referred to as “depth gradient” in Table 1). One additional sampling replicate using 45 mm mesh size was performed at 2 m depths to increase samples of shallow‐spawning large‐sized whitefish. This was carried out on 31 December 2007 in the same locality (referred to as “Supp. Large” in Table 1). For each of the main sampling events, pelagic nets with mesh sizes of 38 and 45 mm were also set at 2–7 m depth, close to the depth gradient location over at least 60 m water depth (referred to as “Pelagic” in Table 1). Sampling of other known spawning site locations within Lake Lucerne was carried out to test for isolation‐by‐distance (IBD) within species (Figure 1; referred to as “Large 1–3,” “Int 1,” “Small 1–3” in Table 1). Gill nets were set at either at 2–5 m depth using 45 mm mesh size or at 40–60 m depth for 26 mm mesh size. One sample of *C. zugensis* was obtained outside of spawning season (referred to as “Small n.s.” in Table 1) and was only included in analyses that did not require structuring by spawning location or spawning depth. Additionally, spawning aggregates of summer‐deep‐spawning *C. nobilis* and of winter‐spawning *C*. sp. “Alpnacherfelchen” (referred to as “Alpnach” in Table 1) were sampled. All nets were set overnight for around 15 hrs.

**Table 1:** Sampling summary for all whitefish caught

Name	Location		Sampling Date	Sample size	*N*_Year 3_	*N*_Small_	*N*_Int._	*N*_Large_	*N*_Genetics_
	North	East							
Depth gradient	47°01′36.37″	8°25′31.56″	19 November–18 December 2007	268	149	107	34	8	149
Supp. Large	47°01′36.37″	8°25′31.56″	31 December 2007	8	6	—	—	6	6
Pelagic	47°01′34.00″	8°24′47.00″	19 November–18 December 2007	66	19	—	14	5	19
Large 1	47°01′44.71″	8°23′42.23″	13 December 2005	41	16	—	—	16	16
Large 2	46°58′31.81″	8°33′05.74″	22 December 2005	30	6	—	—	6	6
Large 3	46°58′24.01″	8°36′29.52″	14 December and 22 December 2005	57	10	—	—	10	10
Int 1	46°58′24.01″	8°36′29.52″	14 December and 22 December 2005	6	6	—	6	—	6
Small 1	47°02′37.04″	8°23′11.80″	19 November and 29 December 2005	9	3	3	—	—	3
Small 2	47°01′40.95″	8°25′42.53″	21 November 2005	25	7	7	—	—	7
Small 3	46°59′53.11″	8°35′05.27″	21 December 2005	63	15	15	—	—	14
Small n.s.	47°02′37.04″	8°23′11.80″	19 November 2005	16	5	5	—	—	5
*C. nobilis*	46°59′25.55″	8°29′10.24″	21 July–02 August 2005	38	n/a	n/a	n/a	n/a	38
Alpnach	46°57′52.11″	8°19′10.49″	02 December 2004	20	n/a	n/a	n/a	n/a	20
Total				647	242	137	54	51	299

Given for each sampling event are the event name, geographic location, date of sampling, number of whitefish caught (sample size), the number of three‐year‐old whitefish that were included in the population genetic analyses (*N*_Year 3_) and the sample sizes corresponding to each standard length (SL) class (*N*_Small_, *N*_Int._, and *N*_Large_) and the number of individuals included in genetic analyses (*N*_Genetics_).

All fish were weighed, had their standard length (SL) measured and had a piece of muscle tissue taken and preserved in absolute ethanol. Ageing was carried out using annual growth rings identified on scales from above the lateral line. Gill‐raker counts for each individual were determined using the first left gill arch. Only sexually ripe or almost ripe fish were used in subsequent analyses (maturation degree 5 or 6; Smolina, 1920), with the exception of the “Small n.s.” sample. A detailed summary of whitefish sampled, sampling date and location for each sampling site is given in Table 1.

### 2.2 DNA extraction and microsatellite amplification

DNA was extracted using a Qiagen^®^ Bio Sprint 96 extraction robot according to the manufacturer's standard extraction protocol (Qiagen, Zug, Switzerland). All fish were genotyped at 10 microsatellite loci: Cocl‐Lav49, Cocl‐Lav61, Cocl‐Lav6, Cocl‐Lav68, Cocl‐Lav10, Cisco‐157, Cocl‐Lav4, Cocl‐Lav45, Cocl‐Lav18 and BWF‐2 following Vonlanthen et al. (2009).

### 2.3 Sampling data analysis

To test for an effect of sampling depth and spawning time on the structuring of ecologically important phenotypic variation among sampled depth gradient whitefish, linear regressions of standard length (SL) and gill‐raker number versus water depth and sampling date of capture were performed in R 3.1.2 (R Core Team 2014). Deviations from a normal distribution in body size (SL) and gill‐raker counts for all depth gradient whitefish were tested using a Shapiro–Wilk test. Where deviations from normality were observed, the fit of a mixture of two or three normal distributions was compared based on Akaike's information criterion corrected for sample size (AIC_c_), using discmixtureprogs v 0.4 (Brewer, 2003). To test whether gaps observed in fish standard length distributions from our catches could be due to body size selectivity of the different gill net mesh sizes employed, skewed normal selectivity curves were estimated for each mesh size using the R package sn and summed to estimate total selectivity across all mesh sizes (Azzalini & Capitanio, 1999; Fujimori & Tokai, 2001). To test whether the deviations from normality of standard length variation within mesh sizes result from the co‐occurrence of differently aged fish of the same species or from the co‐occurrence of more than one species, three mesh selectivity SL classes were defined based on the selectivity curves for each mesh size obtained above. Boundaries between selectivity SL classes were set at the overall standard length where the selectivity curves between two different mesh sizes crossed. Whitefish were assigned to these classes based on individual SL, irrespective of the actual mesh size each fish was caught in. As within‐mesh standard length deviations from normality likely result from the presence of fish from two or more such selectivity SL classes within one mesh size, the age of fish from all three selectivity SL classes was compared using a Mann–Whitney *U*‐test. If deviations from normality were due to the capture of multiple whitefish species with different growth rates within a single mesh size, no age differences would be expected.

The number of SL classes, potentially representing distinct taxon groupings differing in growth rate, was further determined using a dynamic hybrid tree cut (Langfelder, Zhang, & Horvath, 2008), following Lucek, Kristjánsson, Skúlason, and Seehausen (2016). In short, this method is based on a bottom‐up algorithm that first identifies preliminary clusters within a data set, depending on a given minimal cluster size, the distance and distinctiveness of its neighbouring objects and the connectivity of branches within a cluster. In a second step, previously unassigned objects are tested for their proximity to the preliminary clusters. This method is based on tree topology without prior assumptions on the number of inferred clusters, therefore providing an unbiased estimate for the number of clusters that are present. Dynamic hybrid tree cut was first conducted on three‐year‐old whitefish from the depth gradient only, due to the large sample size and unbiased sampling (Fig. S1, Table 1). three‐year‐old individuals from other winter‐spawning whitefish sampling locations were subsequently assigned to the closest matching cluster based on their respective SLs. A dynamic tree cut analysis was further conducted for each remaining age class (ages: 2, 4, 5 and 6), pooling all available individuals within each age group to increase sample sizes. For each analysis, the assumed minimal cluster size was set to 10% of the available individuals.

### 2.4 Sources of population genetic structure

Four variables were tested for their potential contribution in shaping genetic variation along the depth gradient: (i) sampling date (= spawning date), (ii) capture depth, (iii) gill net mesh size and (iv) individual standard length were analysed in an analysis of molecular variance (AMOVA) using arlequin v. 3.5.1.2 (Excoffier & Lischer, 2010) using 10,000 permutations to assess significance. Only Three‐year‐old fish obtained in the depth gradient sampling were used. For the SL‐related AMOVA, whitefish were assigned to one of three groups (small: 160–235 mm, intermediate: 236–320 mm, large: 321–410 mm) suggested by the trimodal frequency distribution of individual standard lengths (see Figure 3a). Fish from other age classes were excluded, because the trimodal pattern was less evident and/or sample sizes were small (Fig. S1). Two distinct population genetic methods were used to explicitly test whether sampled winter‐spawning whitefish diversity in Lake Lucerne was best explained by the existence of two (*K* = 2) or three species (*K* = 3) and to estimate individual assignment probabilities to the identified clusters: Structure v. 2.3.4 (Hubisz, Falush, Stephens, & Pritchard, 2009) and discriminant analysis of principal components (DAPCs) implemented in adegenet 2.01 (Jombart, 2008). Parameters used for structure were the following: 100,000 burn‐in length, 500,000 MCMC chain replicates, admixture model of ancestry and correlated allele frequencies. Twenty structure run iterations were carried out for each *K*. Consensus values for individual assignment probabilities across the independent iterations run at each *K* were calculated in CLUMPAK (Kopelman, Mayzel, Jakobsson, Rosenberg, & Mayrose, 2015). All available winter‐spawning whitefish from the main lake were used for these analyses (Alpnach, *C*. *nobilis* excluded).

three‐year‐old fish caught outside the depth gradient site at different lake‐wide locations were grouped according to their standard length frequency distribution and the SL‐related AMOVA, resulting in three “small‐type” (Small 1–3), three “large‐type” (Large 1–3) and one “intermediate‐type” (Int 1) populations caught in benthic nets, and single populations of both “large‐type” and “intermediate‐type” fish caught in pelagic nets (see Table 1). Genetic differentiation (*F*_ST_) between these populations and the SL class groupings of fish caught on the depth gradient was then calculated in arlequin using 10,000 permutations to assess significance. To test for isolation‐by‐distance (IBD) among three‐year‐old fish caught in different parts of the lake, partial mantel tests were performed with the *vegan* package (Oksanen et al. 2013) in R, correlating individual genetic distances (Smouse & Peakall, 1999) that were calculated in genodive (Meirmans & Van Tienderen, 2004) with geographic distances among sampling locations, while controlling for genetic structure among population groupings based on either individual capture depth or individual standard length. Significance was assessed using 1,000 permutation steps.

### 2.5 Genetic diversity within Lucerne species

Following the pooling of whitefish belonging to the same SL class but showing nonsignificant patterns of genetic differentiation, into distinct species groups, observed (*H*_O_) and expected (*H*_E_) heterozygosities were calculated for each locus within each pooled group in arlequin. Deviations from Hardy–Weinberg equilibrium (HWE) were tested for using Fisher's exact test in genepop v. 4.0 (Rousset, 2008) with 10,000,000 steps in the Markov chain and 10,000 dememorization steps. *F*_IS_ values for each locus in each species and for each species across all loci and number of alleles (*A*_N_) for each species were calculated in fstat v. 2.9.3 (Goudet, 2001). Significance levels of *F*_IS_ and deviations from HWE were corrected for multiple testing using sequential Bonferroni correction (Rice, 1989). Deviations from linkage equilibrium between all pairs of loci for each species were tested for using arlequin. Allelic richness (*A*_R_) and the mean number of private alleles per locus were calculated for each species in ADZE‐1.0 (Szpiech, Jakobsson, & Rosenberg, 2008). Generalized private allelic richness was also calculated between all possible species combinations to measure the number of alleles exclusive to each grouping. This analysis was repeated using only the species groups caught on the depth gradient (*Large*,* Benthic intermediate*,* Small*), to increase the standardized individual sample size (*N* = 34 vs. *N* = 14).

### 2.6 Genetic differentiation among Lake Lucerne species

To estimate neutral marker differentiation among species groups, multilocus pairwise *F*_ST_ values were calculated in arlequin, using 10,000 permutations. A false discovery rate (FDR) correction was subsequently applied to all pairwise comparisons (Benjamini & Hochberg, 1995).

### 2.7 Tests of association between neutral genetic and adaptive phenotypic variation

Using all three‐year‐old individuals sampled from the depth gradient, the correlation between individual genetic differences and individual differences in standard length and gill‐raker number was assessed using Mantel tests with 1,000 permutation steps, using the *vegan* package in R. Partial Mantel tests were further used to test for the same correlation, controlling either for the effects of geographic distance or capture depth.

### 2.8 Genetic relatedness across lakes

To investigate whether historical whitefish introductions might have contributed to contemporary whitefish diversity, genetic comparisons were made between Lake Lucerne taxa and an additional 12 whitefish species representing four major lake radiations within the Alpine radiation (Lakes Brienz/Thun, Constance, Neuchatel and Walen/Zurich). Existing microsatellite genotype data (Bittner, Excoffier, & Largiadèr, 2010; Vonlanthen et al., 2012) were combined with Lake Lucerne genotypes to create a consensus population‐based neighbour‐joining (NJ) tree with 1,000 bootstrap replicates using Cavalli‐Sforza chord distances (*D*_CH_) in phylip v. 3.69 (Felsenstein, 2005). Pairwise *F*_ST_ values between all species were again estimated in arlequin. Additionally, DAPC was used to estimate the overall probability of assignment of Lake Lucerne whitefish to allopatric whitefish populations from other Alpine lakes (Constance, Thun, Brienz, Walen, Neuchatel, Zurich). Pairwise analyses were carried out separately between the constituent species of Lake Lucerne and those of each of the other six lakes. For each pairwise analysis, the lake of origin of each fish was used to define prior groups for the subsequent DAPC. The lowest associated root‐mean‐squared error (RMSE) value obtained following cross‐validation was used to identify the optimal amount of principal components (PCs) retained during DAPC. For the genotyped species from each of these lakes, generalized private allelic richness was again calculated in ADZE‐1.0, to measure the number of alleles exclusive to different multispecies groupings.

## 3 Results

### 3.1 Sampling results

**Figure 2:**
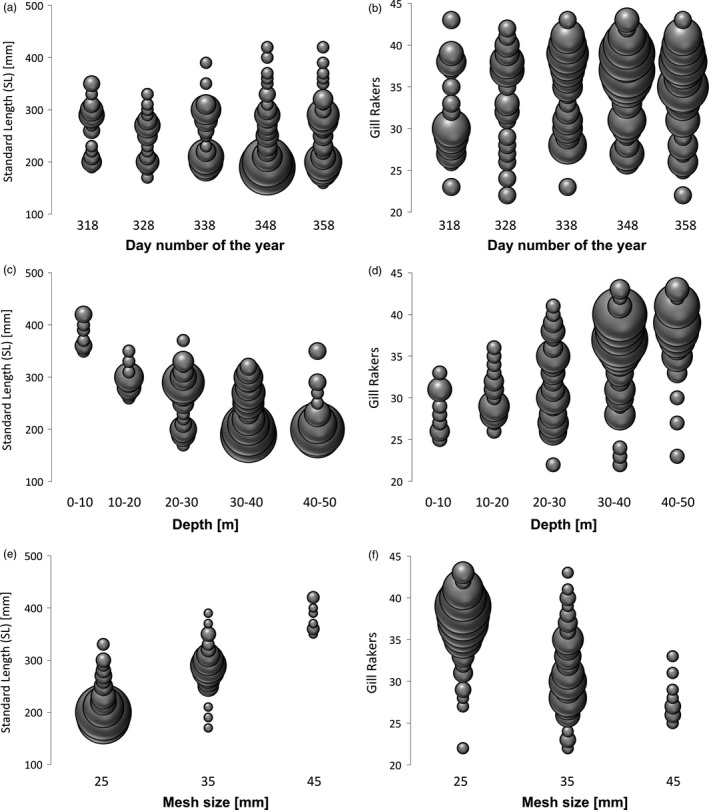
Results of whitefish sampling along a depth and time gradient: (a) standard length (SL) and (b) gill‐raker counts of whitefish caught at five different sampling dates. (c) SL and (d) gill‐raker counts of whitefish caught at different depths. (e) SL and (f) gill‐raker counts of whitefish caught in different mesh sizes

**Figure 3:**
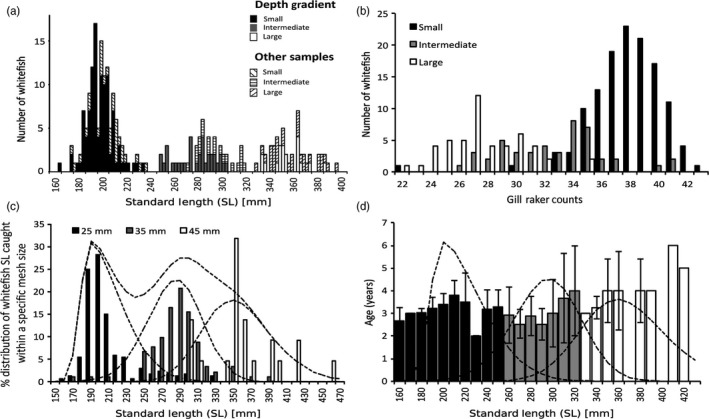
(a) Standard length (SL) histogram of Three‐year‐old, winter‐spawning whitefish from Lake Lucerne. Indicated are the distinct SL clusters as inferred using a dynamic hybrid tree cut of individuals caught on the depth gradient (solid shading). Additional three‐year‐old fish from other sampling locations were subsequently assigned to the closest cluster (hatched shading). (b) Histogram of gill‐raker counts of three‐year‐old fish. Shading indicates individual SL class assignment. (c) Histogram showing the SL distribution of whitefish caught within the same gill net mesh size. Shading denotes the mesh size individuals were caught in. Dashed lines represent the selectivity curves for each gill net mesh size and across all mesh sizes pooled assuming a right‐skewed normal distribution. (d) The average age of whitefish for a given SL class with the corresponding standard deviations. Dashed lines represent the selectivity curves for each mesh size. Black bars are fish with the highest likelihood to be caught in 25 mm, grey bars in 35 mm and white bars in 45 mm mesh sizes

In total, 647 whitefish from Lake Lucerne were analysed (Table 1). Focusing on whitefish sampled along the depth gradient, overall standard length showed a slight but significant decrease (*F*_1,266_ = 5.64, *p *< .05) and gill‐raker counts increased (*F*_1,253_ = 10.52, *p *< .01) with later capture dates (Figure 2a,b). Water depth of capture had a stronger effect on phenotypic variation than capture date, but phenotypic variation showed similar overall trends: SL decreasing (*F*_1,266_ = 205.8, *p *< .001) and gill‐raker counts increasing (*F*_1,253_ = 121.6, *p *< .001) with depth along the gradient (Figure 2c,d). The dynamic hybrid tree cut analysis showed the frequency distribution of standard length at age three to be trimodal, suggesting the occurrence of multiple species along the depth gradient, which differ in growth rate (Figure 3a). The boundaries between the distinct SL peaks were at 235 and 320 mm (Figure 3a). Trimodality was further supported for four‐ to six‐year‐old fish but not for two‐year‐old fish (Figure S1). Gill‐raker counts did not reveal a trimodal pattern, but fish of intermediate body size were also intermediate in gill‐raker counts (Figure 3b). A similar trimodal pattern for standard length could in principle also emerge as a result of mesh size selectivity. Different mesh sizes were biased towards certain SL categories, but fish of the same standard length were still caught in different nets with different mesh sizes (Figures 2e and 3c). Distributions both of SL (nine of 13 comparisons) and gill‐raker counts (eight of 13 comparisons) differed significantly from normality in the majority of mesh sizes, dates and depths (Table S1). For individual standard length, mixture models with either three overlapping normal distributions (six of nine comparisons) or with two to three overlapping normal distributions were equally likely (three of nine comparisons) to explain the SL distribution where it deviated from normality. For gill‐rakers, mixture models with three overlapping normal distributions (one of eight comparisons), two to three overlapping normal distributions (three of nine comparisons) or with only two overlapping normal distributions (four of nine comparisons) best explained the data where it deviated from normality. Results of mixed distribution analysis showed consistent overall trends for both standard length and gill‐rakers: deviations from a normal distribution occurring only at deeper sampling depths (>10 and >20 m, respectively) and within net panels with smaller mesh sizes (<45 and <35 mm). Relative age differed between the three SL classes, defined based on selectivity curves (C_1_: 150–245 mm, C_2_: 246–315 mm, C_3_: 316–430 mm; Figure 3d). Fish from the large SL class tended to be older than fish from the other classes. However, contrary to predictions of age class structure, fish in the intermediate SL class were younger than those in both the small and the large SL classes (Mann–Whitney *U*‐tests: C_1_ with C_2_: *df* = 244, *p* < .001; C_1_ with C_3_: *df* = 181, *p* < .05; C_2_ with C_3_: *df* = 119, *p* < .001; Fig. S1).

### 3.2 Genetic structure

AMOVAs with gradient‐caught individuals assigned to groups based on their SL class explained the highest amount of overall genetic variation, with a global *F*_ST_ of 0.05 (*p* < .001). Using mesh size (global *F*_ST_ = 0.044, *p* < .001) and capture depth (global *F*_ST_ = 0.025, *p* < .001) to group sampled whitefish also explained significant although lower amounts of genetic variation. However, AMOVA with individuals grouped according to their capture date was nonsignificant (global *F*_ST_ = 0.003, *p* = .927). Both population genetic methods of assigning individuals to representative genetic clusters, structure and DAPC, were not effective in discriminating between the three SL classes, but the resulting assignment probabilities for *K* = 2 as well as *K* = 3 were consistent with grouping by standard length (Figs S2 and S3, respectively). Individuals within the large and the small SL classes generally showed relatively strong reciprocal assignment to alternate genetic clusters; however, this became less clear for *K* = 3. Individuals in the intermediate SL class overall showed more ambiguous assignments to specific clusters than those from the other classes. Therefore, subsequent analyses were performed with populations structured by the three SL‐at‐age‐three groupings (small: 160–235 mm, intermediate: 236–320 mm, large: 321–410 mm).

*F*_ST_ values between gradient‐caught populations and those of the same SL class sampled from different geographic locations in Lake Lucerne were nonsignificant (Table S2). One exception to this was the *F*_ST_ value between the intermediate‐sized whitefish caught on the depth gradient and intermediate‐sized whitefish caught in pelagic nets during depth gradient samplings, which was significant (*F*_ST_ = 0.019, *p* = .036). three‐year‐old pelagic intermediate‐sized fish were significantly larger than three‐year‐old benthic intermediate‐sized fish (pelagic $\overset{¯}{x}$ = 289 mm, benthic $\overset{¯}{x}$ = 273 mm; *t*_1,25_ = 3.21, *p* = .003). These populations were thus treated as distinct species groupings for further analyses. The sole allopatric population of intermediate‐sized whitefish that was sampled (Int. 1) could not be assigned to either the benthic or pelagic “intermediate” populations, likely due to its low sample size (*N* = 6), and was excluded from subsequent analyses. Partial Mantel tests revealed no significant IBD when controlling for either individual capture depth (*r* = .015; *p* = .329) or SL at age three (*r* = .030; *p* = .205). Therefore, geographically distinct populations of the small and the large SL classes were pooled (Small 1–3; Large 1–3, plus large individuals caught in the pelagic sample) to be used as species groupings in further analyses. Hereafter, these pooled species groupings are referred to as *Small* and *Large*. Intermediate‐sized fish caught with benthic nets are referred to as *Benthic intermediate* and intermediate‐sized fish caught in the pelagic are referred to as *Pelagic intermediate*. The number of gill‐rakers differed significantly (*t*_1,84_ = 21.83, *p* < .001) between the *Small* ($\overset{¯}{x}$ = 37.8 ± 2.63 *SD*) and *Large* whitefish species ($\overset{¯}{x}$ = 27.7 ± 2.83 *SD*). Gill‐raker counts also differed between each of these whitefish species and *Benthic intermediates* ($\overset{¯}{x}$ = 33.3 ± 3.79 *SD*;* t*_1,51_ = −7.06, *p* < .001 and *t*_1,37_ = 6.3, *p* < .001 for comparisons with *Large* and *Small*, respectively) and *Pelagic intermediates* ($\overset{¯}{x}$ = 32.46 ± 3.04 *SD*;* t*_1,18_ = −5.07, *p* < .001, *t*_1,14_ = 6.13, *p* < .001, respectively). However, gill‐raker counts did not significantly differ between *Benthic intermediates* and *Pelagic intermediates* (*t*_1,28_ = 0.76, *p* = .45).

### 3.3 Genetic diversity within Lucerne species

For population genetic comparisons involving all Lucerne whitefish species (including *Alpnach* whitefish and *C*. *nobilis* in addition to the three taxa present along the depth gradient), no deviations from HWE and no significant *F*_IS_ values were observed after Bonferroni correction (Table 2). Overall, 12 deviations from linkage equilibrium were observed, representing 4.4% of all pairwise comparisons and similar to the amount expected by chance, with no apparent over‐representation of specific loci pairs in the subset found to be significantly out of linkage equilibrium. Allelic richness ranged from 2.95 (*C. nobilis*) to 3.61 (*Benthic intermediate*; Table 2). The mean number of private alleles per locus found within each species ranged from 0.17 (*Benthic intermediate*) to 0.35 (*Alpnach*). For alleles exclusive to all the possible two‐species and three‐species groupings for Lucerne taxa, the highest mean number of private alleles per locus values were found for *Large* + *Benthic intermediate* (0.11) and *Large* + *Benthic intermediate* + *Pelagic intermediate* (0.07) groupings (Table S3). Using only the three pooled species groupings found along the depth gradient, allelic richness remained highest in the *Benthic intermediate* (4.53 vs. 4.37–4.42) and the highest amounts of shared private alleles were again found between the *Large* and *Benthic intermediate* groupings (0.57 vs. 0.16–0.4; Table S3).

**Table 2:** Summary of genetic diversity within whitefish species groupings

Grouping	SL	*N*	*H*_O_	*H*_E_	*p‐*HWE	*A*_R_	*F*_IS_	*p‐F*_IS_	*N*_LD_
Small	160–235 mm	136	0.48	0.50	n.s.	3.36	0.04	n.s.	3
Benthic intermediate	236–320 mm	34	0.54	0.57	n.s.	3.61	0.06	n.s.	1
Pelagic intermediate	236–320 mm	14	0.53	0.58	n.s.	3.47	0.09	n.s.	2
Large	321–381 mm	51	0.57	0.59	n.s.	3.55	0.03	n.s.	2
*Coregonus nobilis*	—	38	0.47	0.47	n.s.	2.95	0.01	n.s.	3
Alpnach	—	20	0.54	0.57	n.s.	3.43	0.05	n.s.	1
Total/overall		293	0.52	0.55		3.40	0.05		12

For each species grouping, we report the standard length range at age three (SL), the sample size (*N*), observed (*H*_O_) and expected heterozygosity (*H*_E_), significance level of deviation from HWE across all loci (*p‐*HWE), allelic richness (*A*_R_), inbreeding coefficient (*F*_IS_) and its significance level (*p‐F*_IS_), number of deviations from linkage equilibrium (*N*_LD_) at a significance level of *p* = .05. Significance levels for HWE and *F*_IS_ are Bonferroni corrected.

### 3.4 Genetic differentiation among Lake Lucerne species

Lucerne species groupings showed significant levels of pairwise genetic differentiation from one another, following FDR multitest correction (*F*_ST_ = 0.019–0.124; Table 3). Pairwise *F*_ST_ values were highest between the *Small* species (corresponding to *C. zugensis*) and the *Large* species (*C*. sp. “Bodenbalchen”; *F*_ST_ = 0.124, *p* < .001). Pairwise *F*_ST_s between *Benthic intermediate* and both the *Small* and *Large* species were relatively similar (*F*_ST_ = 0.028 and 0.029, respectively, *p* < .001). Genetic differentiation between the *Pelagic intermediate* and the *Benthic intermediate* was low but significant (*F*_ST_ = 0.019, *p* < .05). The *Pelagic intermediate* was more strongly differentiated from both the *Small* (*F*_ST_ = 0.064, *p* < .001) and *Large* species (*F*_ST_ = 0.04, *p* < .001), than the *Benthic intermediate* species was from either (Table 3).

**Table 3:** Genetic differentiation between Lake Lucerne whitefish species based on 10 neutral microsatellite loci

	Small	Benthic intermediate	Pelagic intermediate	Large	*Coregonus nobilis*	Alpnach
Small	—	***	***	***	***	***
Benthic intermediate	0.028	—	*	***	**	***
Pelagic intermediate	0.064	0.019	—	***	**	***
Large	0.124	0.029	0.040	—	***	***
*C. nobilis*	0.028	0.036	0.065	0.107	—	***
Alpnach	0.069	0.022	0.040	0.050	0.092	—

We report pairwise *F*_ST_ values (below the diagonal) and the corresponding significance levels indicated, following FDR correction (above the diagonal: *p < .05, **p < .01, ***p < .001).

### 3.5 Tests of association between neutral genetic and adaptive phenotypic variation

Overall, for whitefish assigned to *Small*,* Large* or *Benthic intermediate* species, the phenotypic trait showing the strongest correlation with patterns of individual genetic distance was SL (Mantel test: *r* = .409, *p *< .001), followed by gill‐raker count (Mantel test: *r* = .351, *p *< .001) and capture depth (Mantel test: *r* = .274, *p *< .001). Moreover, both individual SL and gill‐raker counts were strongly correlated (Mantel test: *r* = .694, *p *< .001). Capture depth explained significant residual variation when correcting for the effect of gill‐rakers (partial Mantel test: *r* = .125, *p* < .001) but not SL (partial Mantel test: *r* = .018, *p* = .242), while both SL (*r* = .315, *p* < .001) and gill‐raker number (*r* = .260, *p* < .001) remained significantly associated with pairwise genetic distance when correcting for capture depth. SL (*r* = .394, *p* < .001) and gill‐rakers (*r* = .342, *p* < .001) also remained significant when controlling for the effects of geography.

### 3.6 Genetic relatedness of species across lakes

Neighbour‐joining trees show that whitefish species within the Alpine radiation predominately cluster with other coexisting whitefish species, forming monophyletic lake or connected‐lake radiations (Figure S4). One exception to this pattern is that Lake Constance taxa form a nested grouping within a predominantly Lucerne clade. Here, Lucerne *Pelagic intermediate* is sister taxon to *Coregonus wartmanni* from Lake Constance, although this relationship was weakly supported (<50% bootstrap support). The other Lucerne taxa form two distinct clades consistent with patterns of pairwise neutral differentiation: the *Large* species grouping with *Alpnach* and *Benthic intermediates* (<50%); high gill‐raker species (*Small* and *C. nobilis*) forming a separate grouping (70.5%). Pairwise *F*_ST_s between allopatric whitefish species were generally higher ($\overset{¯}{x}$ = 0.15; range = 0.023–0.33) than those found within Lake Lucerne ($\overset{¯}{x}$ = 0.054; range = 0.019–0.124; Table S4). However, certain allopatric species showed low levels of neutral genetic differentiation from Lake Lucerne taxa, despite a lack of geographic proximity between lakes. *Coregonus zuerichensis* from Lake Zurich showed the overall lowest pairwise *F*_ST_ with a Lake Lucerne species (0.023; *Pelagic intermediate*). *Coregonus candidus* from Lake Neuchatel also showed low levels of genetic differentiation from both intermediate Lucerne species (both 0.027).

**Figure 4:**
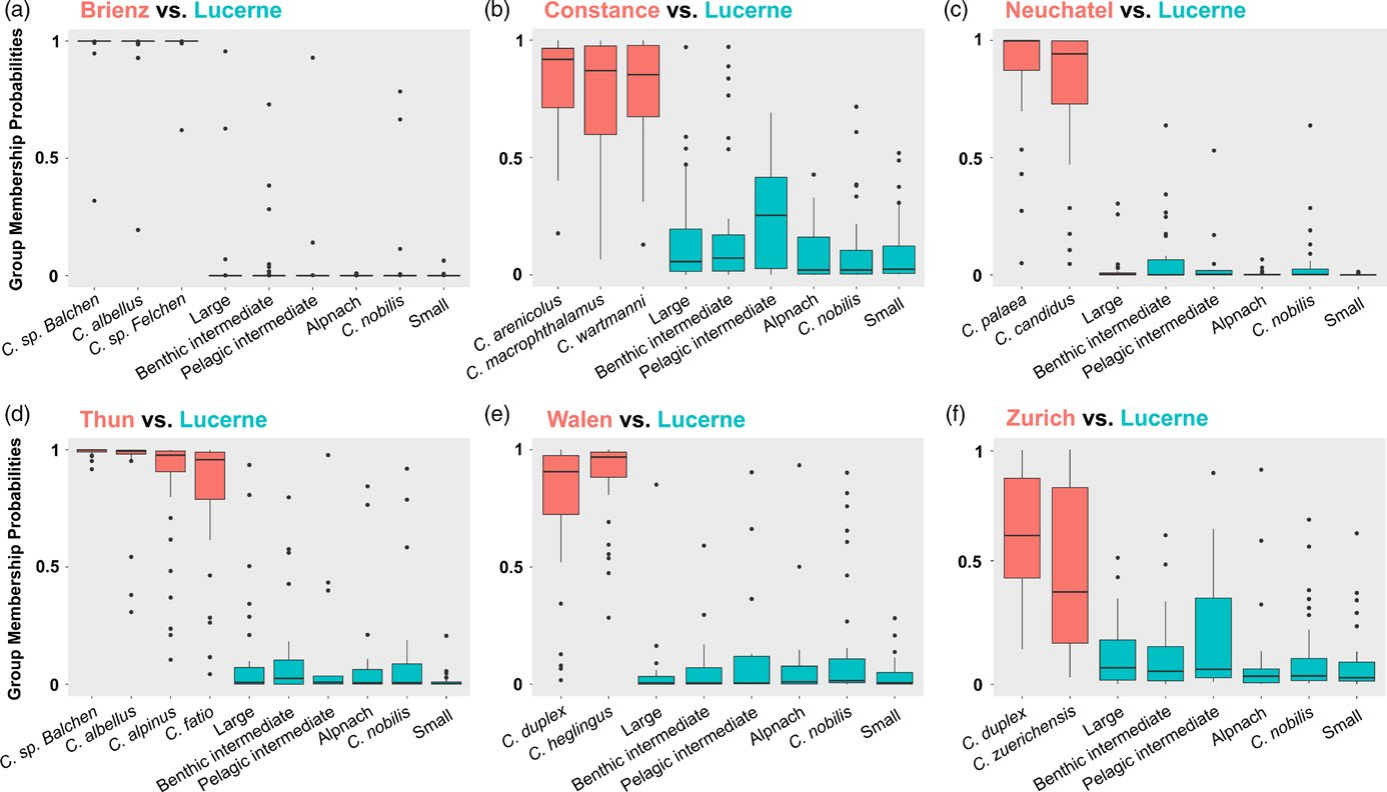
Likelihood of assignment of individuals from sampled whitefish species to allopatric genetic clusters (1) versus a Lucerne cluster (0). Pairwise DAPC comparisons were made using Lake Lucerne whitefish and whitefish from (a) Lake Brienz, (b) Lake Constance, (c) Lake Neuchatel, (d) Lake Thun, (e) Lake Walen, (f) Lake Zurich. Box plots represent the interquartile range around the median assignment probability for each taxon to the non‐Lucerne cluster in each pairwise lake comparison

Pairwise DAPC comparisons between Lake Lucerne and other Alpine lake species flocks varied among species and among lakes in their strength of assignment to one of the two clusters specified (Figure 4). Comparisons involving Brienz, Thun, Neuchatel and Walen generally showed high median (>0.9) assignment probabilities for all species back to their lake of origin (Figure 4a,c–e). The Lake Zurich species showed relatively low median assignment probabilities to their native cluster (*Coregonus duplex* = 0.60; *C*. *zuerichensis* = 0.37), with much broader interquartile ranges than in other lake comparisons. This pattern was not reciprocated by the Lucerne taxa in this comparison, all species showing low median assignment probabilities to the non‐native cluster (0.027–0.069; Figure 4f). In the Lake Constance comparison, *Pelagic intermediate* showed higher median membership probabilities to the Constance flock (0.254) than other Lucerne species (0.021–0.072; Figure 4b).

Including the species from the other Swiss lakes, the highest mean number of shared private alleles per locus in two‐species comparisons involving Lucerne whitefish was for the grouping of *Pelagic intermediate* and allopatric Constance *C. wartmanni* (0.053; Table S5). The second highest amount was shared between sympatric *Benthic intermediates* and the *Large* species (0.04). All other values for comparisons involving at least one Lucerne taxon were considerably lower (≤0.019). For three‐species comparisons involving Lucerne taxa, the highest mean number of private alleles was shared between sympatric *Large*,* Pelagic intermediate* and *Benthic intermediate* species (0.026). However, an allopatric taxa grouping involving Lake Constance *C*. *wartmanni* and *Coregonus macrophthalmus* together with Lucerne *Pelagic intermediates* also had relatively high values (0.017).

## 4 Discussion

By carrying out a detailed analysis of ecological spatial structuring within the endemic whitefish radiation of Lake Lucerne, we demonstrate the importance of environmental gradients for speciation and species coexistence, even at fine spatial scales. We delineate six distinct coexisting whitefish taxa forming an endemic species flock, including the existence of two new whitefish species: *C*. sp. “benthic intermediate” and *C*. sp. “pelagic intermediate.” We also identify aspects of how these complex and fragile evolutionarily young radiations have been impacted by past and present fisheries management. Focusing on whitefish spawning benthically in late autumn/winter, we find correlated patterns of adaptive phenotypic and neutral genetic variation distributed along a benthic depth gradient during the winter‐spawning season. Importantly, the distribution of phenotypic variation along this depth gradient was not continuous, instead consisting of three distinct clusters, with individuals from the different clusters intermingling at intermediate depths. Despite this demographic admixture on the spawning grounds, significant genetic differentiation between phenotypic clusters was maintained, indicating that whitefish making up the three distinct clusters maintain some level of reproductive isolation from one another. In close proximity to the three species found along the sampled depth gradient: *C. zugensis* (“*Small*”), *C*. sp. “benthic intermediate” (“*Benthic intermediate*”), *C*. sp. “Bodenbalchen” (“*Large*”), an additional whitefish species was characterized from pelagic nets: *C*. sp. “pelagic intermediate” (“*Pelagic intermediate*”). For the species found spawning along the depth gradient, we find a strong association of individual genetic variation with growth rate and gill‐raker counts, traits suggested to be evolving under divergent selection in whitefish (Østbye et al., 2006; Præbel et al., 2013; Rogers & Bernatchez, 2007; Vonlanthen et al., 2009). These findings are consistent with predictions of isolation‐by‐adaptation and ecological speciation along clines in our studied depth gradient (Funk, Nosil, & Etges, 2006; Nosil, 2012; Rundle & Nosil, 2005). Despite indications of a non‐native origin to *C*. sp. “pelagic intermediate,” the full extent of how anthropogenic impacts such as eutrophication, stocking and other fisheries management practices have influenced whitefish diversity present in Lake Lucerne remains unclear. The discovery of two previously undescribed species in this study highlights the importance of taxonomically unbiased sampling strategies to assess the whole diversity present in a system: to both understand the evolutionary mechanisms structuring contemporary biodiversity and to better inform conservation and fisheries management plans.

### 4.1 The endemic whitefish diversity of Lake Lucerne

In total, we find empirical support for the presence of six genetically and phenotypically differentiated whitefish species in Lake Lucerne. Multiple axes of divergence appear to structure endemic whitefish species diversity: depth/location of spawning, time/duration of spawning and trophic ecology. Three *Coregonus* species were previously known from Lake Lucerne: the deep‐spawning, small‐bodied, densely rakered *C. zugensis*; the shallow‐spawning, large bodied, sparsely rakered *C*. sp. “Bodenbalchen” (classified under *Coregonus suidteri*); and the recently rediscovered summer‐deep‐spawning, intermediate‐sized, densely rakered *C. nobilis*—an ecomorph unique to Lake Lucerne (Kottelat & Freyhof, 2007). A fourth undescribed species, *C*. sp. “Alpnacherfelchen” (“*Alpnach*”), appears to be restricted to a separate sub‐basin of Lake Lucerne, Lake Alpnach (Hudson et al., 2011; Svarvar & Müller, 1982). More research is required to establish the ecological and evolutionary distinctness of the latter taxon from other Lucerne whitefish. The final two species were previously unknown or poorly characterized and were identified in this study through in‐depth eco‐spatially informed sampling throughout the spawning season and spawning depth range that was known for *C. zugensis* and *C*. sp. “Bodenbalchen.”

Along the sampled spawning depth gradient, we find three distinct whitefish species structured in a phenotypic, likely adaptive (growth rate and gill‐raker counts), and neutral genotypic cline. This depth–habitat‐associated cline did not consist of a continuous phenotypic and genotypic gradation as the frequency distribution of SL of three‐year‐old fish was trimodal. Sampling bias caused by the different gill net mesh sizes alone cannot explain the absence of fish of specific body size required to generate a unimodal standard length distribution along the spawning depth gradient. Also, the age structure of whitefish provided evidence for more than one whitefish species co‐occurring along the depth gradient with small SL class whitefish older than the larger intermediate SL class fish, suggesting the existence of at least two whitefish species differing strongly in their growth rates. Analysis of the structuring of neutral genetic variation along the depth gradient provided the strongest evidence for multiple coexisting whitefish species spawning in close proximity. Grouping whitefish into three distinct classes according to the trimodal standard length distribution explained the highest amount of genetic variation. Utilizing the SL classes as distinct population units, *Large* and *Small* SL class whitefish could be assigned to shallow‐spawning *C*. sp. “Bodenbalchen” and deep‐spawning *C. zugensis*, respectively. While phenotypic and genetic differentiation was greatest between *C. zugensis* and *C*. sp. “Bodenbalchen” at the extremes of the depth gradient, both species were significantly differentiated in allele frequencies and gill‐raker counts from the whitefish comprising the intermediate SL class, alongside clear differences in growth rate and the existence of alleles private to each taxa: strong evidence for the existence of a third, as yet undescribed, species: *C*. sp. “benthic intermediate,” which spawns benthically at intermediate depths in Lake Lucerne.

The second previously unknown whitefish species consisted of individuals caught in pelagic nets, in close proximity to the depth gradient. These whitefish were intermediate‐sized, although with a higher growth rate than *C*. sp. “benthic intermediate.” The significant genetic differentiation between these pelagic whitefish and other whitefish forms spawning in close spatial proximity, including the phenotypically similar *C*. sp. “benthic intermediate,” suggests these individuals may constitute a new Lucerne whitefish species: *C*. sp. “pelagic intermediate.”

### 4.2 Evidence for ecological speciation along an environmental gradient

If the current eco‐spatial structuring of whitefish diversity along the spawning depth gradient is in any way indicative of the diversity present within the ancestral population at the onset of speciation, this may have facilitated divergence. Here, any significant association of ancestral genotypes with aspects of the environment along the depth gradient could bias mating encounter rates among individuals with different trait values, facilitating the origin of linkage disequilibrium between unlinked genes coding for these traits, and hence enable the origin of divergently adapted and reproductively isolated species through clinal speciation (Gavrilets, 2004). One aspect of our study system that is not captured by classical models of clinal speciation is that the observed depth cline appears only to be limited to the spawning season. A key aspect of many clinal speciation models is that dispersal is limited along the environmental gradient, decreasing gene flow and increasing the fitness consequences of localized selection regimes (Doebeli & Dieckmann, 2003; Heinz, Mazzucco, & Dieckmann, 2009; Kawata et al., 2007). Yet, whitefish outside the spawning season disperse over much greater distances than the geographic extent of the spawning gradient and indeed this study found co‐occurrence of multiple species within certain depths during the spawning season. How has reproductive isolation evolved/been maintained along the Lucerne depth gradient, given the potentially high levels of dispersal? One answer to this may be that dispersal is not random with respect to the phenotype of the individuals and their chosen environment through matching habitat choice (Edelaar, Siepielski, & Clobert, 2008). With increasing depth in lacustrine environments, abiotic components of the environment such as temperature, oxygen concentration, light intensity and spectral composition change rapidly alongside concomitant changes in biotic components (Seehausen et al., 2008). Coupled changes in these factors will create localized divergent selection regimes at different locations along lacustrine depth gradients. Matching habitat choice would result in spatial clustering of phenotypically similar individuals along these environmental gradients and if spawning location/timing were also governed by these phenotype–fitness interactions, matching habitat choice would facilitate assortative mating and the beginnings of reproductive isolation. Support for this scenario comes from parallel patterns of spawning habitat divergence in multiple independent lake flocks within the Alpine whitefish radiation: small, densely gill‐rakered zooplanktivores spawning deeper than coexisting larger, sparsely gill‐rakered, zoobenthos feeding ecomorphs (Steinmann, 1950; Vonlanthen et al., 2012). The ecological factors driving predictable patterns of spawning depth/time segregation among coexisting whitefish ecomorphs remain an understudied aspect of speciation and adaptive radiation in this species complex.

While speciation from an eco‐spatially structured ancestral population is theoretically less constrained than from one approaching panmixis, strong disruptive selection regimes and/or strong assortative mating would likely still be required to drive population divergence, especially given the high potential for gene flow (Doebeli & Dieckmann, 2003; Gavrilets, 2004; Nosil, 2012). Indeed, several lines of evidence support an ecological speciation scenario via the action of divergent natural selection for the origin of Lake Lucerne whitefish: (i) our study confirms previous works (Douglas, Brunner, & Bernatchez, 1999; Hudson et al., 2011) in showing that similar ecomorphs have arisen repeatedly across different lake flocks in the Alpine whitefish radiation. (ii) Significant structuring of neutral genetic and adaptive phenotypic variation was observed along an ecological gradient (water depth), whereas nonsignificant isolation‐by‐distance was uncovered between spatially distant but phenotypically similar populations within Lake Lucerne, strong evidence for the primacy of selective rather than neutral processes in the generation of intralacustrine whitefish diversity. (iii) Species diverging along the depth gradient show significant differences in traits related to niche utilization: gill‐raker number and growth rate. Gill‐rakers are projections on the gill arches thought to act as cross‐flow filters to improve prey handling and retention (Sanderson, Cheer, Goodrich, Graziano, & Callan, 2001). Higher densities of gill‐rakers along the gill arch have been shown to increase feeding efficiency on zooplankton in Alpine whitefish (Roesch, Lundsgaard‐Hansen, Vonlanthen, Taverna, & Seehausen, 2013). Alpine whitefish with lower gill‐raker densities, on the other hand, have been shown to be more efficient at foraging for large benthic prey items (Lundsgaard‐Hansen, Matthews, Vonlanthen, Taverna, & Seehausen, 2013). These predictable differences in feeding efficiency are suggestive of fitness trade‐offs in gill‐raker number between the respective niches of coexisting Alpine whitefish species, corroborated by genetic evidence that within‐lake patterns of gill‐raker count variation are driven by divergent natural selection regimes in the Alpine and other whitefish radiations (Hudson et al., 2013; Præbel et al., 2013; Rogers & Bernatchez, 2007; Vonlanthen et al., 2009). Whitefish growth rate is a complex physiological trait impinging on many other aspects of the overall phenotype such as body shape. Interspecific differences in growth rate among whitefish species have been shown to be heritable (Lundsgaard‐Hansen et al., 2013; Rogers & Bernatchez, 2007), and differences in growth rate have been shown to affect foraging ability in different aquatic niches in Alpine whitefish, where larger bodied whitefish are more efficient at exploiting benthic food resources (Lundsgaard‐Hansen et al., 2013). Additional roles for growth rate in whitefish adaptive divergence and speciation may include escaping size‐related predation windows differing among niches (Kahilainen & Lehtonen, 2002) and size assortative mate choice (Foote & Larkin, 1988). Again, genetic evidence supports the role of divergent natural selection in driving patterns of interspecific variation in growth rate in different whitefish radiations (Rogers & Bernatchez, 2007; Vonlanthen et al., 2009). While both gill‐raker number and growth rate are clearly ecologically important traits involved in adaptive divergence and potentially speciation, the frequency distributions of adult body size, indicative of growth rate, better reflected individual genetic differences among sampled depth gradient whitefish than gill‐raker counts. Further work is required to establish the exact trophic differences among the three species caught along the gradient, but body size may interact with gill‐raker counts by changing the overall size of the gill arch, altering gill‐raker density irrespective of the actual gill‐raker number: heightening species’ foraging performance in their respective habitats. (iv) Despite the observed demographic admixture (as opposed to obvious genetic admixture) between the three whitefish species on their spawning grounds, low but significant genetic differentiation between them is maintained, suggesting that these whitefish species are reproductively isolated. Overall, the above observations are consistent with isolation‐by‐adaptation and ecological speciation along an environmental gradient (Doebeli & Dieckmann, 2003; Endler, 1977; Nosil, 2012; Schluter, 2009).

### 4.3 Anthropogenic impacts on whitefish diversity: implications for management

Two interacting anthropogenic impacts may have had profound effects on shaping endemic Lake Lucerne whitefish diversity: (i) eutrophication and (ii) stocking, both of allochthonous and autochthonous whitefish. Given the relative evolutionary youth of the Alpine whitefish radiation as a whole, mating barriers between the constituent species will be largely based on behavioural and/or extrinsic pre‐ and postzygotic isolating mechanisms rather than intrinsic genomic incompatibilities (Eckmann, 2015; Vonlanthen et al., 2012; Woods et al., 2009). In some Swiss lakes, anthropogenic eutrophication has led to a complete collapse of whitefish diversity over the past 60 years due to an interaction between negative population growth and speciation reversal (Vonlanthen et al., 2012). While levels of eutrophication were not as severe in Lake Lucerne, nutrient enrichment at its peak was likely high enough to impact the available spawning depth range and therefore potentially increase overlap and gene flow among spawning whitefish species, potentially contributing to the relatively low levels of genetic divergence currently seen among the depth gradient whitefish species (Vonlanthen et al., 2012). One potential outcome of human‐mediated gene flow is the origin of *C*. sp. “benthic intermediate” through homoploid hybrid speciation via secondary contact and introgression between *C. zugensis* and *C*. sp. “Bodenbalchen” (Mavárez & Linares, 2008). *C*. sp. “benthic intermediate” is both intermediate in its spawning habitat (assuming this is equivalent to its capture depth), growth rate and gill‐raker counts, and shows equivalent levels of genetic divergence between the two other depth gradient species, consistent with this theory. However, levels of private allelic richness suggest that *C*. sp. “benthic intermediate” is an independent evolutionary taxon and not merely a hybrid of recent origin.

Stocking of non‐native whitefish from different Alpine lakes might have increased intralacustrine whitefish species diversity in Lake Lucerne, at least in the short term. Douglas and Brunner (2002) include a population from Lake Lucerne labelled only as “Blaufelchen.” This population groups closest in their study to Lake Constance taxa and another taxon of recognized allochthonous origin, *C*. *fatioi* from Lake Thun (Hudson et al., 2011). Blaufelchen is the local name for *C. wartmanni*, an intermediate‐sized whitefish endemic to Lake Constance, with relatively high gill‐raker counts (mean = 34.5; Vonlanthen et al., 2012). The *C*. sp. “pelagic intermediate” individuals were caught aggregating in the open water and were sexually ripe. This suggests that this species may spawn in the pelagic, a spawning habitat previously only recorded from *C. wartmanni* (Kottelat & Freyhof, 2007). Alongside phenotypic similarities: DAPC, microsatellite‐based trees and shared private allele analyses support a genetic affinity between *C*. sp. “pelagic intermediate” and *C. wartmanni* individuals from Lake Constance. Historical records also exist of whitefish fry from Lake Constance being stocked into Lake Lucerne (Steinmann, 1950). This evidence is consistent with *C. wartmanni* being a progenitor of *C*. sp. “pelagic intermediate” via anthropogenic introduction. Under this scenario, subsequent hybridization with Lucerne's native whitefish species would have reduced genetic and phenotypic differentiation between this form and other co‐occurring whitefish species. Importantly, despite the close proximity of their sampling locations and phenotypic similarities, *C*. sp. “benthic intermediate” was significantly genetically differentiated from *C*. sp. “pelagic intermediate,” suggesting some degree of reproductive isolation between these species, and showed no indication of being of non‐native origin itself. The long‐term consequences of this stocking on native Lucerne whitefish diversity remain unknown. Given the apparent phenotypic similarity of the two intermediate‐sized species, potential outcomes include either the introduced species or *C*. sp. “benthic intermediate” ecologically outcompeting one another or both collapsing through introgression into a hybrid swarm, causing one or both species’ extinction. A similar situation to this has occurred in the lakes of the Pasvik River system on the Norwegian/Russian border with the invasion of a congeneric zooplankton specialist (*C*. *albula*) into lakes containing native European whitefish species. This has resulted in hybridization between the invasive species and native whitefish in certain lakes (Kahilainen et al., 2011). In other lakes, competitive exclusion of native densely rakered whitefish species by the invasive has occurred, combined with massive introgression and the loss of phenotypic and genetic differentiation between the previously distinct densely rakered and large sparsely rakered whitefish species (Bhat et al., 2014). Alternatively, neutral models of coexistence (Leibold & McPeek, 2006), potentially followed by ecological character displacement or specialization within the shared niche, buttressed by the nonoverlap of spawning habitats between these species could allow the persistence of both species. While increased sampling of *C*. sp. “benthic intermediate” and *C*. sp. “pelagic intermediate” populations across Lake Lucerne would be helpful to draw exact inferences about their origins, ecology and co‐distribution within the lake, further research on these species within the context of the local radiation may provide new insight into the factors that determine the extent of adaptive radiations and the hypothesis of diversity‐dependent slowdown of speciation rates (Schluter, 2000).

Given the fine‐scale partitioning of the spawning depth habitat in Lake Lucerne, a more insidious threat to intralacustrine whitefish diversity might be the supplemental stocking of native whitefish. Carried out during the spawning season, ripe adult fish are caught on their spawning grounds, their gametes stripped and mixed, and the resulting fertilized eggs are raised to fingerlings before being released. This practice is carried out in many Swiss lakes, and these stocked whitefish may comprise a large proportion of a species’ population (e.g., up to 83% of *C*. *wartmanni* in Lake Constance; Eckmann, 2012), despite a lack of evidence for any resultant long‐term yield benefits especially in healthy lacustrine ecosystems. Alongside impairing natural recruitment, especially in nontarget whitefish species, and imposing inadvertent artificial sexual selection on target species (Eckmann, 2012), supplemental stocking may increase rates of introgressive hybridization as demographically mixed catches of individuals from different species but of broadly similar appearance (e.g., older/larger *C. zugensis*, younger/smaller *C*. sp. “benthic intermediate”) are accidentally treated as a single stock for crossing. The increase in observed intralacustrine complexity highlighted in our study will require the development of more sophisticated fisheries and conservation management plans to maintain whitefish diversity for stakeholders and posterity (Lankau, Jørgensen, Harris, & Sih, 2011; Lapointe et al., 2014).

## 5 Conclusions

Through spatially and ecologically informed sampling, our study has revealed that currently Lake Lucerne holds at least six distinct whitefish species, comprising one of the most diverse intralacustrine species flocks within the Alpine whitefish radiation and the wider *C*. *lavaretus* species complex globally. Along a depth gradient during winter‐spawning time, we find a previously unknown species: *C*. sp. “benthic intermediate”, currently distinct in gill‐raker counts, growth rate and allele frequencies from both known whitefish species that spawn in close proximity. The nondiscrete nature of phenotypic variation among whitefish species spawning along the depth gradient makes *C*. sp. “benthic intermediate” vulnerable to increased gene flow via fisheries management techniques such as supplemental stocking, where the true extent of coexisting whitefish diversity may be underestimated. Moreover, the tight spatial packing of species on the spawning gradient, coupled to the dependence of spawning niche segregation on the persistence of fine‐scale depth‐related differences in the lacustrine environment, makes these species especially vulnerable to increased gene flow via changes in the physicochemical habitat characteristics, such as those associated with changes in primary productivity. A sixth distinct species, *C*. sp. “pelagic intermediate,” differing in growth rate and reproductive ecology from *C*. sp. “benthic intermediate,” may be wholly or partially of non‐native origin, the result of whitefish stocking between lakes and further evidence of anthropogenic impacts on Lucerne whitefish biodiversity.

## Data Archiving Statement

Raw data used in this study are available at Data Dryad Digital Repository: http://doi:10.5061/dryad.7g2c9.
