# Supplementary material for: Managing cryptic biodiversity: Fine‐scale intralacustrine speciation along a benthic gradient in Alpine whitefish (Coregonus spp.)
Source: Evol Appl. 2016 Dec 20;10(3):251–66. doi: 10.1111/eva.12446 (PMC5322408; doi:10.1111/eva.12446)
Supplement: Supplementary file 1 [file EVA-10-251-s001.docx]

**Managing cryptic biodiversity: fine-scale intralacustrine speciation along a benthic gradient in Alpine whitefish (*Coregonus* spp.)**

Alan G. Hudson, Baenz Lundsgaard-Hansen, Kay Lucek, Pascal Vonlanthen and Ole Seehausen

**Supplementary Information**

Fig. S1: Standard length (SL) histograms of sampled winter spawning whitefish from Lake Lucerne for each measured age class. Cluster based assignments using a dynamic hybrid tree cut method are indicated, with individuals from unbiased depth gradient caught samples (“Depth”) and targeted samplings (“Other”) shaded differently. Samples that could not be assigned to a specific SL cluster are shaded black. As SL within age class 2 was found not to be multimodal, the histogram for this age class is shaded differently. Also given are individual sample sizes for the different sampling schemes within each age class.


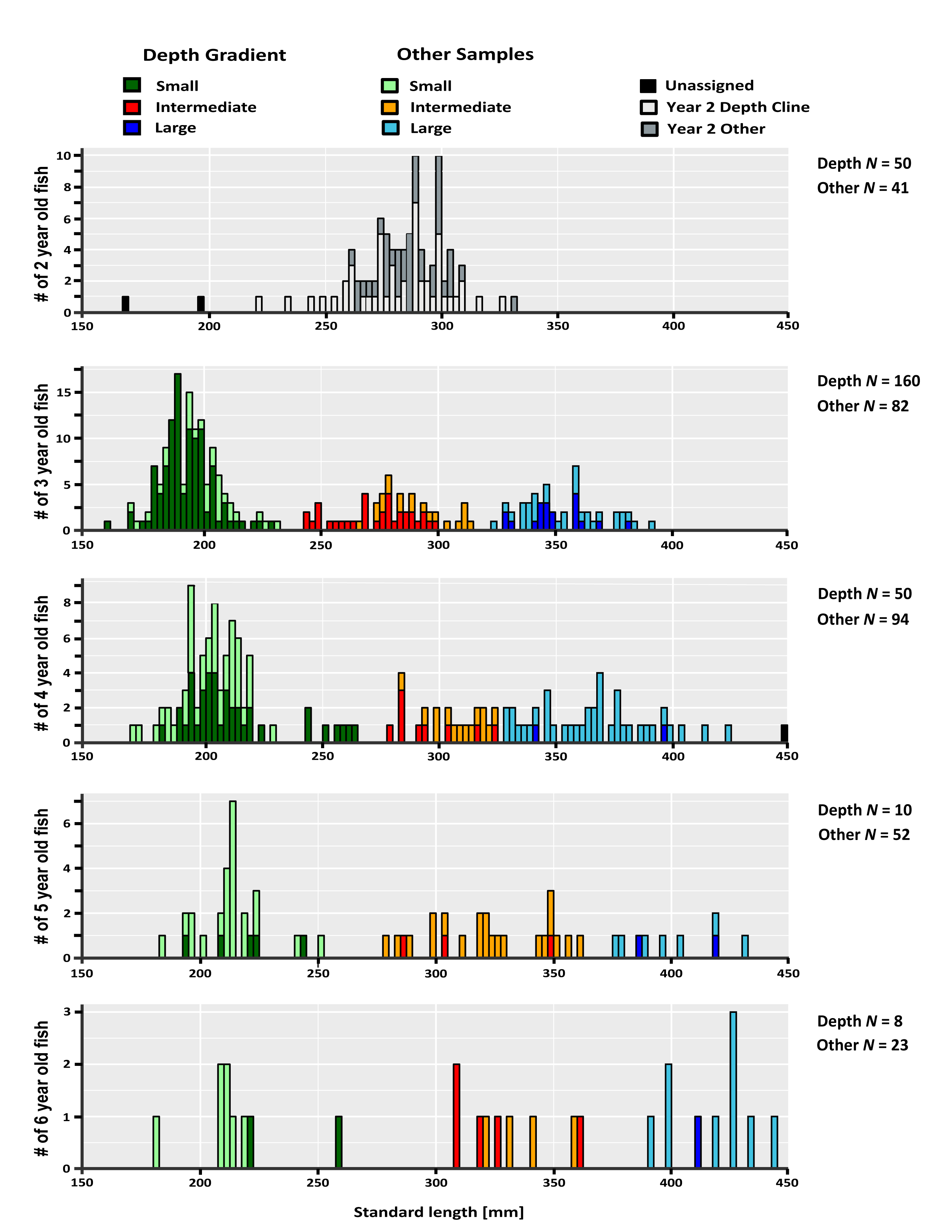


Fig. S2: Structure plots showing posterior individual assignment likelihoods for Lake Lucerne winter-spawning whitefish, for either K=2 or K=3 genetic clusters. All available winter-spawning whitefish from the main lake were included in this analysis, but results are only shown for three year old fish. Individuals are sorted according to their standard length (SL), with increasing size from left to right.

Fig. S3: DPAC plots showing posterior individual assignment probabilities for Lake Lucerne winter-spawning whitefish, for either K=2 or K=3 genetic clusters. All available winter-spawning whitefish from the main lake were included in this analysis, but results are only shown for three year old fish. Individuals are sorted according to their standard length (SL), with increasing size from left to right.

Fig. S4: Population-based tree using Cavalli-Sforza distances based on 10 microsatellite markers, showing genetic relatedness among Alpine whitefish species. Microsatellite data was available for fish from Lake Lucerne (LU), Lake Neuchatel (NE), Lake Constance (CO), Lake Zurich (ZU), Lake Walen (WA), Lake Thun (TH) and Lake Brienz (BR). Colours used for species names, tree branches and representative phenotypes indicate the sampled lakes or lake systems (red: Lake Lucerne; green: Lake Constance; blue: Lake Thun/Brienz; purple: Lake Neuchatel; black: Lake Zurich/Walen). Bootstrap support values >50 % are displayed.


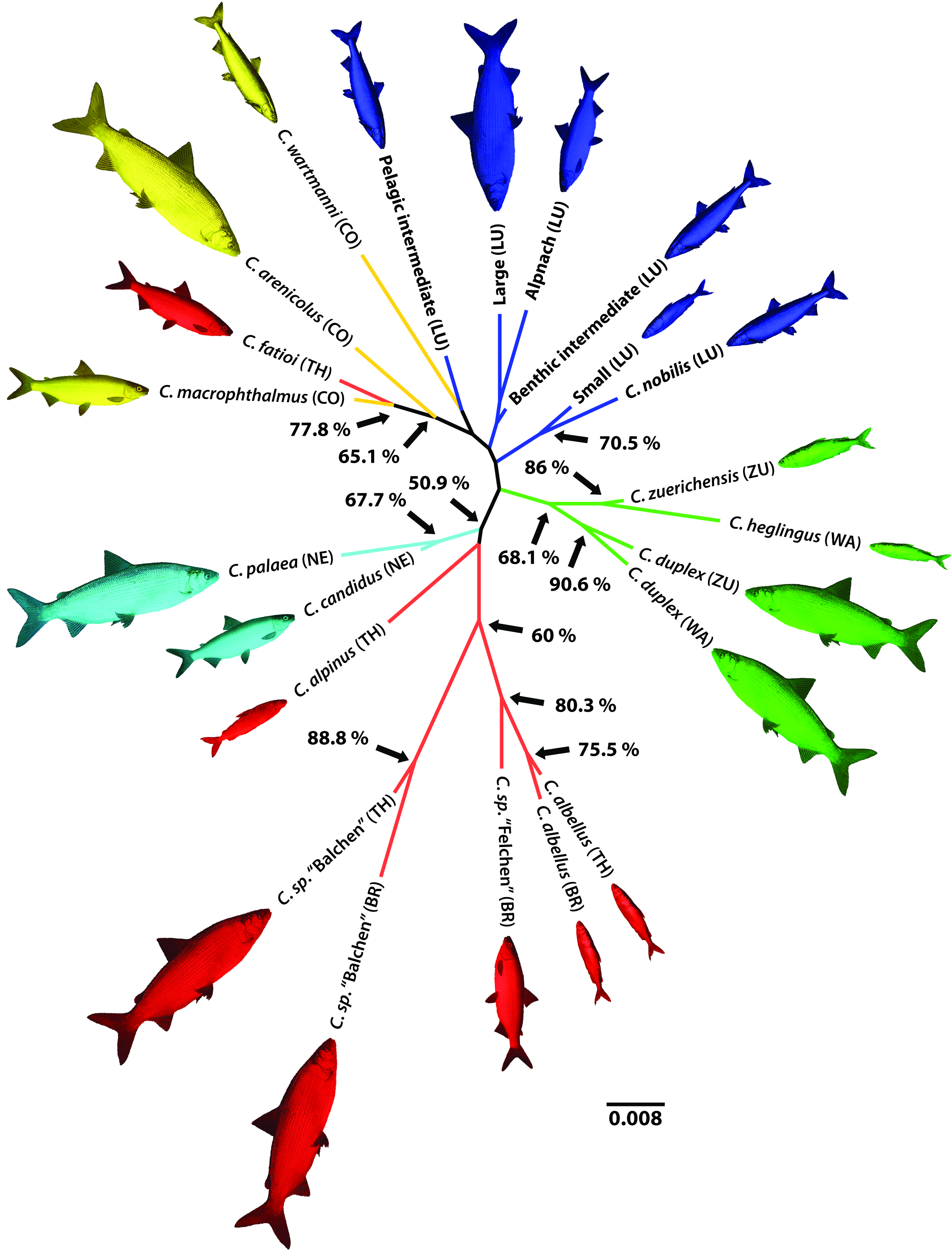


Table S1: Summary of tests for normal distribution and distribution mixture models for depth gradient standard length and gill-raker data. The data was analysed using the three potential grouping variables (mesh size, date, depth) for fish caught on the depth gradient (Grouping variable), each divided into dependent classes (Grouping classes). Shown for each grouping: the summary statistics for the Shapiro-Wilk’s test for normality (W), the degrees of freedom (d.f.) and the corresponding *P*-value. If the distribution significantly differed from a normal distribution, the fit based on Akaike’s Information criterion for single (AICc1), mixtures of two (AICc2), or three (AICc3) normal distributions are given. If ∆AIC_23_ (AICc2- AICc3) is greater than 4, then a mixed model of three normal distributions is more likely. If it is smaller than -4 and if ∆AIC_12_ (AICc1- AICc2) is larger than 4, then a mixture of two normal distributions explain the data best. If ∆AIC_23_ (AICc2- AICc3) is between 4 and -4, a mixture of two or three normal distributions is equally likely.

| Trait | Grouping variable | Grouping classes | W | d.f. | p-value | AICc1 | AICc2 | AICc3 | ∆AIC_12_ | ∆AIC_23_ |
| --- | --- | --- | --- | --- | --- | --- | --- | --- | --- | --- |
| Body size | Mesh size | 25mm | 0.783 | 168 | **<0.001** | 3197 | 773 | 771 | 2424 | 2 |
|  |  | 35mm | 0.927 | 91 | **<0.001** | 3247 | 820 | 807 | 2427 | 13 |
|  |  | 45mm | 0.928 | 9 | 0.466 | - | - | - | - | - |
|  | Date | 19.11.2007 | 0.914 | 27 | **<0.05** | 1387 | 267 | 267 | 1121 | -1 |
|  |  | 26.11.2007 | 0.955 | 27 | 0.289 | - | - | - | - | - |
|  |  | 05.12.2007 | 0.858 | 51 | **<0.001** | 2769 | 489 | 472 | 2279 | 18 |
|  |  | 11.12.2007 | 0.786 | 89 | **<0.001** | 5210 | 879 | 840 | 4331 | 39 |
|  |  | 18.12.2007 | 0.909 | 74 | **<0.001** | 4615 | 760 | 726 | 3855 | 34 |
|  | Depth | 2m | 0.953 | 10 | 0.700 | - | - | - | - | - |
|  |  | 10m | 0.958 | 20 | 0.505 | - | - | - | - | - |
|  |  | 20m | 0.908 | 56 | **<0.001** | 2799 | 528 | 531 | 2271 | -4 |
|  |  | 30 m | 0.884 | 117 | **<0.001** | 5340 | 1070 | 1051 | 4270 | 19 |
|  |  | 40m | 0.663 | 65 | **<0.001** | 2479 | 549 | 535 | 1931 | 14 |
| Gill-rakers | Mesh size | 25mm | 0.896 | 157 | **<0.001** | 851 | 670 | 677 | 181 | -8 |
|  |  | 35mm | 0.975 | 89 | 0.086 | - | - | - | - | - |
|  |  | 45mm | 0.913 | 9 | 0.335 | - | - | - | - | - |
|  | Date | 19.11.2007 | 0.908 | 27 | **<0.05** | 209 | 143 | 152 | 66 | -9 |
|  |  | 26.11.2007 | 0.921 | 27 | **<0.05** | 223 | 159 | 155 | 64 | 4 |
|  |  | 05.12.2007 | 0.927 | 49 | **<0.01** | 366 | 261 | 251 | 106 | 10 |
|  |  | 11.12.2007 | 0.922 | 81 | **<0.001** | 561 | 396 | 402 | 165 | -6 |
|  |  | 18.12.2007 | 0.938 | 71 | **<0.01** | 515 | 366 | 367 | 149 | -1 |
|  | Depth | 2m | 0.929 | 10 | 0.441 | - | - | - | - | - |
|  |  | 10m | 0.933 | 20 | 0.179 | - | - | - | - | - |
|  |  | 20m | 0.970 | 49 | 0.240 | - | - | - | - | - |
|  |  | 30m | 0.899 | 114 | **<0.001** | 770 | 547 | 550 | 223 | -3 |
|  |  | 40m | 0.851 | 62 | **<0.001** | 368 | 274 | 280 | 94 | -6 |

Table S2: Genetic differentiation between Lake Lucerne whitefish populations based on 10 microsatellite loci. We report pairwise *F*_ST_ values (below the diagonal) and the corresponding *P*-value (above the diagonal). Given also are sample sizes (N).

|  | **N** | **1** | **2** | **3** | **4** | **5** | **6** | **7** | **8** | **9** | **10** | **11** | **12** | **13** | **14** | **15** |
| --- | --- | --- | --- | --- | --- | --- | --- | --- | --- | --- | --- | --- | --- | --- | --- | --- |
| **1** Small depth gradient | 107 | - | ns | ns | ns | ns | *** | *** | ** | *** | ** | *** | *** | *** | *** | *** |
| **2** Small 1 | 3 | 0.01 | - | ns | ns | ns | ns | ns | ns | ** | ns | *** | ns | * | * | t |
| **3** Small 2 | 7 | 0.00 | 0.02 | - | ns | ns | * | *** | * | *** | ** | *** | *** | *** | ** | ** |
| **4** Small 3 | 14 | 0.00 | 0.04 | 0.01 | - | ns | * | *** | t | *** | * | *** | *** | *** | *** | ns |
| **5** Small ns | 5 | 0.01 | 0.00 | 0.02 | 0.02 | - | ns | ns | t | *** | ns | *** | t | ** | * | t |
| **6** Benthic intermediate | 34 | 0.03 | 0.02 | 0.04 | 0.02 | 0.01 | - | * | ns | ** | ns | *** | t | ** | ** | *** |
| **7** Pelagic intermediate | 14 | 0.06 | 0.02 | 0.08 | 0.06 | 0.02 | 0.02 | - | ns | ** | ns | ** | ns | ** | ** | *** |
| **8** Int 1 | 6 | 0.04 | 0.04 | 0.05 | 0.03 | 0.03 | 0.00 | 0.00 | - | ns | ns | ns | ns | ns | ns | ** |
| **9** Large depth gradient | 14 | 0.13 | 0.11 | 0.14 | 0.11 | 0.10 | 0.03 | 0.04 | 0.00 | - | ns | ns | ns | ns | ** | *** |
| **10** Large pelagic | 5 | 0.07 | 0.00 | 0.08 | 0.08 | 0.02 | 0.00 | 0.02 | 0.00 | 0.03 | - | t | ns | ns | * | ** |
| **11** Large 1 | 16 | 0.14 | 0.13 | 0.14 | 0.11 | 0.09 | 0.03 | 0.05 | 0.00 | 0.00 | 0.04 | - | ns | ns | ** | *** |
| **12** Large 2 | 6 | 0.12 | 0.07 | 0.12 | 0.11 | 0.05 | 0.03 | 0.02 | 0.00 | 0.00 | 0.01 | 0.00 | - | ns | ** | *** |
| **13** Large 3 | 10 | 0.16 | 0.13 | 0.15 | 0.15 | 0.12 | 0.05 | 0.05 | 0.00 | 0.00 | 0.04 | 0.00 | 0.00 | - | *** | *** |
| **14** Alpnach | 20 | 0.07 | 0.07 | 0.06 | 0.05 | 0.05 | 0.02 | 0.04 | 0.00 | 0.04 | 0.05 | 0.05 | 0.07 | 0.06 | - | *** |
| **15** *C. nobilis* | 38 | 0.03 | 0.05 | 0.06 | 0.01 | 0.03 | 0.04 | 0.07 | 0.05 | 0.12 | 0.09 | 0.12 | 0.10 | 0.15 | 0.09 | - |

*** p<0.001; ** p<0.01; * p<0.05; t = p<0.1; n.s. = non significant

Table S3: Shared private allele richness among different groupings of Lake Lucerne whitefish (A_PR_). Shared private allele richness is given for all sampled whitefish species (All, rarefied population size = 14) and for just three year old fish caught during depth gradient sampling (Cline (N_MAX_ = 34)). Also presented are the variance (σ^2^) and standard deviation (σ_X_).

| **Species 1** | **Species 2** | **Species 3** | **All (N_MAX_ = 14)** | | | **Cline (N_MAX_ = 34)** | | |
| --- | --- | --- | --- | --- | --- | --- | --- | --- |
|  |  |  | **A_PR_** | **σ^2^** | **σ_X_** | **A_PR_** | **σ^2^** | **σ_X_** |
| Small | - | - | 0.330 | 0.132 | 0.115 | 0.689 | 0.600 | 0.245 |
| Large | - | - | 0.260 | 0.045 | 0.067 | 0.460 | 0.239 | 0.155 |
| Benthic intermediate | - | - | 0.170 | 0.024 | 0.049 | 0.384 | 0.121 | 0.110 |
| Pelagic intermediate | - | - | 0.218 | 0.094 | 0.097 | - | - | - |
| *C. nobilis* | - | - | 0.249 | 0.110 | 0.105 | - | - | - |
| Alpnach | - | - | 0.346 | 0.163 | 0.128 | - | - | - |
| Small | Large | - | 0.035 | 0.001 | 0.009 | 0.157 | 0.028 | 0.053 |
| Small | Benthic intermediate | - | 0.056 | 0.007 | 0.027 | 0.395 | 0.167 | 0.129 |
| Small | Pelagic intermediate | - | 0.038 | 0.001 | 0.012 | - | - | - |
| Small | *C. nobilis* | - | 0.059 | 0.021 | 0.046 | - | - | - |
| Small | Alpnach | - | 0.032 | 0.003 | 0.016 | - | - | - |
| Large | Benthic intermediate | - | 0.105 | 0.019 | 0.044 | 0.572 | 0.181 | 0.135 |
| Large | Pelagic intermediate | - | 0.056 | 0.004 | 0.019 | - | - | - |
| Large | *C. nobilis* | - | 0.012 | 0.001 | 0.007 | - | - | - |
| Large | Alpnach | - | 0.061 | 0.006 | 0.024 | - | - | - |
| Benthic intermediate | Pelagic intermediate | - | 0.069 | 0.007 | 0.027 | - | - | - |
| Benthic intermediate | *C. nobilis* | - | 0.041 | 0.007 | 0.027 | - | - | - |
| Benthic intermediate | Alpnach | - | 0.055 | 0.009 | 0.030 | - | - | - |
| Pelagic intermediate | *C. nobilis* | - | 0.034 | 0.004 | 0.020 | - | - | - |
| Pelagic intermediate | Alpnach | - | 0.032 | 0.005 | 0.022 | - | - | - |
| *C. nobilis* | Alpnach | - | 0.017 | 0.001 | 0.012 | - | - | - |
| Small | Large | Benthic intermediate | 0.029 | 0.002 | 0.012 | - | - | - |
| Small | Large | Pelagic intermediate | 0.025 | 0.001 | 0.010 | - | - | - |
| Small | Large | *C. nobilis* | 0.011 | 0.000 | 0.005 | - | - | - |
| Small | Large | Alpnach | 0.013 | 0.000 | 0.003 | - | - | - |
| Small | Benthic intermediate | Pelagic intermediate | 0.028 | 0.001 | 0.012 | - | - | - |
| Small | Benthic intermediate | *C. nobilis* | 0.058 | 0.025 | 0.050 | - | - | - |
| Small | Benthic intermediate | Alpnach | 0.016 | 0.000 | 0.005 | - | - | - |
| Small | Pelagic intermediate | *C. nobilis* | 0.017 | 0.000 | 0.006 | - | - | - |
| Small | Pelagic intermediate | Alpnach | 0.009 | 0.000 | 0.004 | - | - | - |
| Small | *C. nobilis* | Alpnach | 0.005 | 0.000 | 0.002 | - | - | - |
| Large | Benthic intermediate | Pelagic intermediate | 0.073 | 0.008 | 0.029 | - | - | - |
| Large | Benthic intermediate | *C. nobilis* | 0.009 | 0.000 | 0.004 | - | - | - |
| Large | Benthic intermediate | Alpnach | 0.058 | 0.005 | 0.021 | - | - | - |
| Large | Pelagic intermediate | *C. nobilis* | 0.011 | 0.000 | 0.005 | - | - | - |
| Large | Pelagic intermediate | Alpnach | 0.063 | 0.018 | 0.042 | - | - | - |
| Large | *C. nobilis* | Alpnach | 0.004 | 0.000 | 0.002 | - | - | - |
| Benthic intermediate | Pelagic intermediate | *C. nobilis* | 0.009 | 0.000 | 0.003 | - | - | - |
| Benthic intermediate | Pelagic intermediate | Alpnach | 0.024 | 0.002 | 0.013 | - | - | - |
| Benthic intermediate | *C. nobilis* | Alpnach | 0.013 | 0.001 | 0.007 | - | - | - |
| Pelagic intermediate | *C. nobilis* | Alpnach | 0.003 | 0.000 | 0.001 | - | - | - |

Table S4: Genetic differentiation between Alpine whitefish populations based on 10 neutral microsatellite loci. We report species name (Species), lake of origin (LU=Lucerne, CO=Constance, BR=Brienz, TH=Thun, NE=Neuchatel, WA=Walen, ZU=Zurich) and sample sizes (N), pairwise *F*_ST_ values (below the diagonal) and the corresponding p-value (above the diagonal). See Vonlanthen *et al*. (2012) for a map.

| **Species** | **Lake** | **N** | **1** | **2** | **3** | **4** | **5** | **6** | **7** | **8** | **9** | **10** | **11** | **12** | **13** | **14** | **15** | **16** | **17** | **18** | **19** | **20** | **21** | **22** |
| --- | --- | --- | --- | --- | --- | --- | --- | --- | --- | --- | --- | --- | --- | --- | --- | --- | --- | --- | --- | --- | --- | --- | --- | --- |
| **1 Small** | LU | 20 | - | *** | *** | *** | *** | *** | *** | *** | *** | *** | *** | *** | *** | *** | *** | *** | *** | *** | *** | *** | *** | *** |
| **2 *C. nobilis*** | LU | 38 | 0.03 | - | *** | *** | *** | *** | *** | *** | *** | *** | *** | *** | *** | *** | *** | *** | *** | *** | *** | *** | *** | *** |
| **3 Large** | LU | 14 | 0.12 | 0.11 | - | *** | *** | *** | *** | *** | *** | *** | *** | *** | *** | *** | *** | *** | *** | *** | *** | *** | *** | *** |
| **4 Benthic intermediate** | LU | 34 | 0.03 | 0.04 | 0.03 | - | * | *** | *** | *** | *** | *** | *** | *** | *** | *** | *** | *** | *** | *** | *** | *** | *** | *** |
| **5 Pelagic intermediate** | LU | 51 | 0.06 | 0.07 | 0.04 | 0.02 | - | *** | *** | *** | *** | *** | *** | *** | *** | *** | *** | *** | *** | *** | *** | *** | * | *** |
| **6 Alpnach** | LU | 136 | 0.07 | 0.09 | 0.05 | 0.02 | 0.04 | - | *** | *** | *** | *** | *** | *** | *** | *** | *** | *** | *** | *** | *** | *** | *** | *** |
| **7 *C. arenicolus*** | CO | 47 | 0.10 | 0.13 | 0.09 | 0.05 | 0.08 | 0.08 | - | *** | *** | *** | *** | *** | *** | *** | *** | *** | *** | *** | *** | *** | *** | *** |
| **8 *C. macrophthalmus*** | CO | 46 | 0.12 | 0.12 | 0.07 | 0.05 | 0.08 | 0.10 | 0.05 | - | *** | *** | *** | *** | *** | *** | *** | *** | *** | *** | *** | *** | *** | *** |
| **9 *C. wartmanni*** | CO | 58 | 0.13 | 0.14 | 0.14 | 0.09 | 0.06 | 0.15 | 0.12 | 0.13 | - | *** | *** | *** | *** | *** | *** | *** | *** | *** | *** | *** | *** | *** |
| **10 *C. sp.* "Felchen"** | BR | 62 | 0.20 | 0.18 | 0.14 | 0.14 | 0.14 | 0.18 | 0.23 | 0.20 | 0.19 | - | *** | *** | *** | *** | *** | *** | *** | *** | *** | *** | *** | *** |
| **11 *C. albellus*** | BR | 94 | 0.21 | 0.21 | 0.22 | 0.20 | 0.22 | 0.23 | 0.31 | 0.28 | 0.31 | 0.13 | - | *** | *** | *** | *** | *** | *** | *** | *** | *** | *** | *** |
| **12 *C. sp.* "Balchen"** | BR | 57 | 0.31 | 0.33 | 0.23 | 0.24 | 0.24 | 0.29 | 0.30 | 0.26 | 0.18 | 0.14 | 0.33 | - | *** | *** | *** | *** | *** | *** | *** | *** | *** | *** |
| **13 *C. alpinus*** | TH | 75 | 0.16 | 0.15 | 0.08 | 0.09 | 0.10 | 0.11 | 0.12 | 0.11 | 0.16 | 0.18 | 0.27 | 0.26 | - | *** | *** | *** | *** | *** | *** | *** | *** | *** |
| **14 *C. albellus*** | TH | 47 | 0.23 | 0.21 | 0.19 | 0.19 | 0.19 | 0.23 | 0.30 | 0.26 | 0.27 | 0.05 | 0.04 | 0.24 | 0.25 | - | *** | *** | *** | *** | *** | *** | *** | *** |
| **15 *C. sp.* "Balchen"** | TH | 34 | 0.26 | 0.27 | 0.16 | 0.17 | 0.18 | 0.23 | 0.22 | 0.16 | 0.14 | 0.14 | 0.30 | 0.03 | 0.18 | 0.23 | - | *** | *** | *** | *** | *** | *** | *** |
| **16 *C. fatioi*** | TH | 32 | 0.10 | 0.14 | 0.05 | 0.06 | 0.08 | 0.10 | 0.09 | 0.03 | 0.14 | 0.15 | 0.26 | 0.21 | 0.11 | 0.22 | 0.12 | - | *** | *** | *** | *** | *** | *** |
| **17 *C. candidus*** | NE | 43 | 0.07 | 0.06 | 0.06 | 0.03 | 0.03 | 0.08 | 0.08 | 0.07 | 0.10 | 0.13 | 0.19 | 0.24 | 0.07 | 0.17 | 0.17 | 0.07 | - | *** | *** | *** | *** | *** |
| **18 *C. palaea*** | NE | 37 | 0.16 | 0.15 | 0.06 | 0.07 | 0.09 | 0.13 | 0.12 | 0.07 | 0.15 | 0.12 | 0.22 | 0.18 | 0.09 | 0.18 | 0.12 | 0.06 | 0.04 | - | *** | *** | *** | *** |
| **19 *C. heglingus*** | WA | 100 | 0.11 | 0.11 | 0.19 | 0.12 | 0.14 | 0.14 | 0.22 | 0.21 | 0.20 | 0.22 | 0.21 | 0.32 | 0.25 | 0.22 | 0.28 | 0.24 | 0.17 | 0.23 | - | *** | *** | *** |
| **20 *C. duplex*** | WA | 98 | 0.14 | 0.13 | 0.11 | 0.10 | 0.08 | 0.13 | 0.15 | 0.12 | 0.16 | 0.21 | 0.22 | 0.28 | 0.09 | 0.22 | 0.21 | 0.14 | 0.08 | 0.13 | 0.17 | - | *** | *** |
| **21 *C. zuerichensis*** | ZU | 20 | 0.05 | 0.06 | 0.10 | 0.04 | 0.02 | 0.08 | 0.09 | 0.08 | 0.09 | 0.16 | 0.20 | 0.26 | 0.13 | 0.19 | 0.18 | 0.11 | 0.05 | 0.11 | 0.08 | 0.05 | - | *** |
| **22 *C. duplex*** | ZU | 20 | 0.11 | 0.11 | 0.07 | 0.05 | 0.07 | 0.08 | 0.12 | 0.09 | 0.15 | 0.15 | 0.16 | 0.26 | 0.10 | 0.15 | 0.18 | 0.12 | 0.07 | 0.09 | 0.12 | 0.04 | 0.05 | - |

*** p<0.001; ** p<0.01; * p<0.05

Table S5: Shared private allele richness among different species groupings of whitefish from different Swiss lakes (A_PR_). Only presented are the comparisons for each Lucerne species for which the highest A_PR_ values were found, for each level of taxa grouping up to a maximum of four species. Where comparisons involve species sampled from other lakes, the lake of origin is reported (LU=Lucerne, CO=Constance, BR=Brienz, TH=Thun, NE=Neuchatel, WA=Walen, ZU=Zurich). Also presented are the variance (σ^2^) and standard deviation (σ_X_).

| **Species 1** | **Species 2** | **Species 3** | **Species 4** | **N_MAX_ = 14** | | |
| --- | --- | --- | --- | --- | --- | --- |
|  |  |  |  | **A_PR_** | **σ^2^** | **σ_X_** |
| Small (LU) | Pelagic intermediate (LU) | - | - | 0.019 | 0.001 | 0.011 |
| Large (LU) | Benthic intermediate (LU) | - | - | 0.040 | 0.005 | 0.025 |
| Benthic intermediate (LU) | Large (LU) | - | - | 0.040 | 0.005 | 0.025 |
| Pelagic intermediate (LU) | *C*. *wartmanni* (CO) | - | - | 0.053 | 0.020 | 0.050 |
| *C. nobilis* (LU) | *C*. *fatio* (TH) | - | - | 0.019 | 0.003 | 0.019 |
| Alpnach (LU) | *C*. *duplex* (ZU) | - | - | 0.017 | 0.002 | 0.016 |
| Small (LU) | *C. candidus* (NE) | *C*. *duplex* (ZU) | - | 0.013 | 0.001 | 0.013 |
| Large (LU) | Benthic intermediate (LU) | Pelagic intermediate (LU) | - | 0.026 | 0.005 | 0.024 |
| Benthic intermediate (LU) | Large (LU) | Pelagic intermediate (LU) | - | 0.026 | 0.005 | 0.024 |
| Pelagic intermediate (LU) | Benthic intermediate (LU) | Large (LU) | - | 0.026 | 0.005 | 0.024 |
| *C. nobilis* (LU) | Pelagic intermediate (LU) | *C. zuerichensis* (ZU) | - | 0.008 | 0.000 | 0.005 |
| Alpnach (LU) | *C. zuerichensis* (ZU) | *C. heglingus* (WA) | - | 0.017 | 0.001 | 0.013 |
| Small (LU) | *C. zuerichensis* (ZU) | *C. heglingus* (WA) | *C*. *duplex* (ZU) | 0.009 | 0.001 | 0.009 |
| Large (LU) | Small (LU) | Benthic intermediate (LU) | Pelagic intermediate (LU) | 0.005 | 0.000 | 0.004 |
| Benthic intermediate (LU) | Large (LU) | Small (LU) | Pelagic intermediate (LU) | 0.005 | 0.000 | 0.004 |
| Pelagic intermediate (LU) | *C*. *wartmanni* (CO) | *C*. *macrophthalmus* (CO) | *C*. *fatio* (TH) | 0.010 | 0.001 | 0.010 |
| *C. nobilis* (LU) | Small (LU) | Benthic intermediate (LU) | *C*. *sp*. **"**Felchen**"** (BR) | 0.005 | 0.000 | 0.004 |
| Alpnach (LU) | *C. zuerichensis* (ZU) | *C. heglingus* (WA) | *C*. *wartmanni* (CO) | 0.009 | 0.001 | 0.009 |
